# Gut-heart axis at high altitude: a dynamic mediator from hypoxic dysbiosis to adaptive cardioprotection

**DOI:** 10.3389/fmicb.2026.1861538

**Published:** 2026-06-22

**Authors:** Chuankai Shi, Yuan Gao, Li Zhang, Changxu Chen, Lisong Su, Kepeng Ren, Zhecheng Liu, Jiangwei Liu

**Affiliations:** 1 Department of Graduate School, Xinjiang Medical University, Urumqi, China; 2 Key Laboratory of Special Environmental Medicine of Xinjiang, General Hospital of Xinjiang Military Command, Urumqi, China; 3 School of Basic Medical Sciences, Xinjiang Medical University, Urumqi, China; 4 Department of Outpatient, Urumqi Municipal First People's Hospital, Urumqi, Xinjiang, China

**Keywords:** adaptive remodeling, cardiovascular diseases, gut microbiota, high altitude, hypobaric hypoxia, inflammatory response, metabolic disorders, microbiome-targeted interventions

## Abstract

High-altitude hypoxia severely disrupts physiological homeostasis and markedly increases cardiovascular disease (CVD) risk through mechanisms that remain incompletely understood. Emerging evidence regards the gut microbiota as a crucial dynamic regulator within the gut-heart axis, constructing a bridge between the environmental hypoxic stress and the cardiovascular outcomes. This review has summarized the dynamic changes of the gut microbiota in high-altitude environments, from acute dysregulation to adaptive remodeling. We systematically delineate the pathogenic mechanisms whereby acute microbial imbalance drives CVD: at the metabolic level, there is a reduction in the production of short-chain fatty acids (SCFAs), accumulation of trimethylamine N-oxide (TMAO), buildup of hypoxia-induced energy metabolism intermediates (lactic acid and succinic acid), and dysregulation of secondary bile acid metabolism. At the immune inflammatory level, impaired intestinal barrier leads to lipopolysaccharide (LPS) translocation, combined with hypoxia-inducible factor-1α (HIF-1α) overexpression, collectively promoting the development of atherosclerosis, hypertension, and heart failure. The adaptive remodeling reduces vascular injury by enhancing myocardial energy metabolism mediated by SCFA, strengthening the intestinal barrier, regulating anti-inflammatory immunity, stabilizing blood pressure, and also reprogramming uric acid metabolism, thereby playing a role in cardiac protection. Finally, we propose microbiome-targeted intervention strategies, including high-fiber dietary modulation, probiotic/prebiotic/synbiotic supplementation, fecal microbiota transplantation, and metabolite-directed therapies, which provides new theoretical basis and precise therapeutic targets for the prevention of cardiovascular diseases in high-altitude environments.

## 1 Introduction

High-altitude environments pose severe challenges to human physiological homeostasis due to their unique extreme climatic characteristics, including hypobaric hypoxia, cold temperatures, intense ultraviolet radiation, and drastic fluctuations in diurnal temperature (Nian et al., 2018). Hypobaric hypoxia is the core initiating factor that causes a sequence of physiological and pathological alterations in the human body. It acts together with other complicated factors to bring a challenge to the efficiency of body oxygen transportation, the stability of energy metabolism, and the functional reserve of many systems (Cai et al., 2026; Bernardi et al., 2006). In the whole world, over 80 million people permanently reside in places with altitudes above 2,500 m, mainly in areas like the Qinghai-Tibet Plateau, the Andes Mountains, and the East African Plateau (Tremblay and Ainslie, 2021). Furthermore, with the rapid expansion of high-altitude tourism, mining, and research expeditions, over 100 million lowlanders ascend to high altitudes annually via air or land routes, encountering acute hypoxic stress (Xu et al., 2025). According to the related materials, for those who ascend rapidly to high-altitude regions without earlier adaptation, the occurrence of acute mountain sickness increases greatly, though not in a linear manner with the height above sea level. For example, at approximately 3,500 m, the occurrence rate can exceed 25%, and at altitudes above 6,000 m, it generally exceeds 50% (Meier et al., 2017). The clinical manifestations include non-specific symptoms such as headache, nausea, and lassitude; In extremely serious situations, this illness can develop toward life-endangering high-altitude pulmonary edema (HAPE) and high-altitude cerebral edema (HACE) (Armstrong, 2020; Gatterer et al., 2024). This great group of people with great danger makes the prevention and treatment of sicknesses connected with high altitude very important in the whole world’s public health.

The cardiovascular system is a central component of the body’s response to hypoxic stress. Acute high-altitude exposure can triggers a series of pathophysiological changes, including excessive sympathetic nervous system activation, raised pulmonary artery pressure, and myocardial oxygen supply imbalance (Richalet et al., 2024). These acute changes may participate in or aggravate the pathophysiological process of acute mountain sickness, clinically manifesting as palpitations and chest tightness. For patients with pre-existing cardiovascular disease, this superimposed physiological stress greatly raises the risk of exacerbation of their underlying conditions, such as causing angina, leading to decompensated heart failure, or triggering arrhythmias, as well as the risk of acute cardiovascular events (Hansen and Sander, 2003; Parati et al., 2018; Naeije, 2024). Long-term or repeated high-altitude exposure significantly increases the risk of various cardiovascular diseases, including hypertension, myocardial ischemia, heart failure, and pulmonary arterial hypertension (Richalet et al., 2024; Aggarwal et al., 2025). A large cohort study involving 67,275 patients with hypertension showed that, compared with patients residing at low altitudes, those living long-term at altitudes >2,500 m had a 46% increased risk of cardiovascular mortality (Xia et al., 2024), suggesting that chronic high-altitude exposure increases the burden of cardiovascular disease. The occurrence of these cardiovascular events has, traditionally, been ascribed to metabolic reprogramming, oxidative stress damage, vascular endothelial dysfunction, and strengthened systemic or local inflammation reactions, which are mediated by the activation of hypoxia-inducible factor (HIF) pathways (Semenza, 2009; Pham et al., 2021; Zhao et al., 2023). However, the upstream regulatory signals of these pathological processes and their integrated mechanisms remain to be further clarified.

The gut microbiota, a dynamic microbial ecosystem that contains trillions of symbiotic microorganisms, including bacteria, archaea, viruses, and fungi, which live inside the gastrointestinal tract, has recently been acknowledged as a central hub regulating environmental adaptation and disease susceptibility. By means of their metabolites (such as short-chain fatty acids (SCFAs), bile acids, and trimethylamine N-oxide (TMAO)) and microbial components (such as lipopolysaccharides (LPS)), they engage in extensive bidirectional communication with the host at the metabolic, immune, and neuroendocrine levels. This deeply affects the energy metabolism balance of the host, the completeness of the intestinal barrier, and systemic inflammation, playing a critical role in the occurrence and development of many kinds of metabolic and chronic diseases (Wu et al., 2021; Liu et al., 2025a). Notably, the high-altitude hypoxic environment is a key driver shaping the structure and function of the host gut microbiota: acute exposure is often with the feature of gut bacterial imbalance, including the proliferation of opportunistic pathogens, depletion of protective microbiota, and the changes in microbial metabolic functions; whereas after long-term adaptation, the structure of microbial community gradually carries out reorganization, exhibiting distinct adaptive phenotypes, suggesting that the gut microbiota is not merely a passive responder to hypoxic damage but an active regulator of high-altitude adaptation (Zhao et al., 2025; Ma X. et al., 2025). Recent research has gradually revealed that gut microbiota plays a dual role in cardiovascular health at high altitudes. On the one hand, microbiota dysbiosis exacerbates vascular endothelial dysfunction through pathways such as endotoxin translocation mediated by intestinal barrier damage, the release of pro-inflammatory factors, and the accumulation of TMAO, and is associated with many cardiovascular dysfunctions, including heart failure, thrombosis, atherosclerosis, and hypertension (Wu and Chiou, 2021). On the other hand, the gut microbiota, following adaptive remodeling, produces heart-protective effects through mechanisms that include increased SCFAs production, improved uric acid metabolism, and adjustment of immune balance (Chen Q. et al., 2025). This dual nature positions the gut microbiota as a dynamic mediator linking high-altitude environmental stressors to cardiovascular disease outcomes. However, most existing studies have mainly inspected unidirectional associations between “hypoxia and gut microbiota” or “gut microbiota and cardiovascular disease,” lacking a systematic combination of the adjustment axis through which high-altitude environments affect the cardiovascular system via the gut microbiota. In particular, the role of the gut microbiota as an upstream regulatory hub has not yet received sufficient attention in studies on cardiovascular disease at high altitudes. This review aims to systematically summarize the dynamic evolution rules of the gut microbiota in high-altitude environments, conduct deep analysis on the pathogenic mechanisms that microbial imbalance causes cardiovascular disease, make clear the cardiovascular protection functions that are mediated by adaptive changes of the microbial group, and furthermore explore prevention and treatment plans that target the gut microbiota, thereby providing new theoretical foundations and intervention targets for the precise prevention and treatment of cardiovascular diseases at high altitudes.

## 2 Dynamic changes in the gut microbiome in high-altitude environments

### 2.1 Temporal responses of the gut microbiota

When individuals or experimental animals from low-altitude environments are rapidly exposed to high-altitude conditions, which include low air pressure, hypoxia, low temperatures, and intense radiation, the inherent homeostasis of the gut microbiome can be disrupted within hours to days, leading to overall disruptions in gut microbiota diversity, species composition, and functional metabolism (Ma Y. et al., 2025). However, when the time of exposure increases, the gut microbiota does not remain in a state of imbalance but gradually carries out adaptive rebuilding under continuous environmental selective pressure (Su et al., 2024a). This temporal change is both a central component of stress response-mediated cardiovascular damage in high-altitude environments and the microbiological basis for the protective effects of adaptation against cardiovascular disease (Table 1). The changes in gut microbiota diversity act as the key indicators to assess this dynamic process. Among these, *α*-diversity reflects species richness and evenness within the gut microbiota, serving as a measure of microbial ecosystem stability, and *β*-diversity quantifies the overall differences in microbial community structure across different samples; it gives a direct reflection of the reshaping effects that a high-altitude environment has on the whole microbial structure (Andreu-Sánchez et al., 2025). Current research has proven that alterations in α-diversity exhibit significant time dependence: short-term acute exposure for 24 h can lead to increased α-diversity (e.g., Shannon and Simpson indices) (Wang F. et al., 2022), while if the exposure is prolonged and continues for several weeks, it will bring a notable reduction (Wang R. et al., 2022). This indicates that the response of gut microbiota to high-altitude environments may experience a dynamic process, which is from acute stress compensation to chronic maladaptation. In contrast, changes in β-diversity are more persistent and pronounced. Within several days after acute exposure, one can see that the microbial structure has an obvious deviation from the baseline level of lowland areas, and this kind of community structure reorganization still exists even after people come back to low-altitude places, suggesting that the high-altitude environment induces long-term remodeling of the gut microbiome (Ma X. et al., 2025).

**Table 1:** Dynamic shifts of gut microbiota during acute exposure and long-term adaptation to high-altitude environments.

Study subjects	Study design	Changes in major microbial communities following acute exposure	Long-term adaptation to changes in the dominant microbial community	References
406 healthy men	Fecal samples were collected from participants at three time points: G-I (baseline, 800 m), G-II (7 days, 4,500 m), and G-III (after a 3-month stay at 4,500 m, followed by a 2-week return to 800 m); high-throughput 16S rDNA Illumina HiSeq sequencing was employed (relative abundance)	F/B ratio ↓ Escherichia/Shigella ↑ Ruminococcus ↑ Oscillibacter ↑ Faecalibacterium ↓ Roseburia ↓ Bifidobacterium↓ Blautia↓ Collinsella↓	Partial recovery: F/B ratio Return to baseline: Faecalibacterium Roseburia↑ Clostridium ↑ Continuous increase: Prevotella Lachnospiraceae incertae sedis Dialister	Ma X. et al. (2025)
393 healthy men	Divided into 6 groups based on their history of living in high-altitude regions: Han1k, Han4k_4d, Han4k_6d, Han4k (over 3 months), Han4k_b3m, and Tibetan4k; fecal samples were sequenced for the 16S rRNA gene (relative abundance)	F/B ratio ↓ Bifidobacterium ↓	F/B ratio ↓ Roseburia ↑ Butyricimonas↑ Bacteroidetes↑ Acinetobacter↓ Haemophilus↓ Klebsiella ↓ Veillonella↓ Sarcina↑	Jia et al. (2020)
610 healthy men	Divided into 6 groups based on altitude, length of residence, and ethnicity: Han1k_HP, Han1k_KP, Han4k_1w, Han4k_6m, Han4k_d3m, and Tibetan4k_NP; fecal samples were collected and subjected to shotgun metagenomic sequencing using the Illumina HiSeq 2500 (relative abundance)	*Escherichia coli*↓ *Klebsiella pneumoniae*↓ Shigella↓ Ruthenibacterium lactatiformans↑ *Clostridium leptum*↑	F/B ratio↓ *Prevotella copri*↑ Bacteroides↑ *Parabacteroides distasonis*↑ *Escherichia coli*↓ *Klebsiella pneumoniae*↓ Shigella↓ Kluyvera ascorbate↓	Han et al. (2024)
7 mountaineers	Exposed at altitudes above 5,000 m; 7 sampling time points were established, and fresh fecal samples were collected continuously for 29 days; fluorescent *in situ* hybridization (FISH) was used for quantitative detection (absolute quantification)	Bifidobacterium↓ Gamma subdivision of Proteobacteria↑ *Escherichia coli*↑ *Klebsiella pneumoniae*↑	-	Kleessen et al. (2005)
30 male Sprague Dawley (SD) rats	SD rats were randomly divided into 3 groups: Experimental group: antibiotic pretreatment + transplantation of high-altitude mouse and rabbit fecal microbiota; Control group 1: antibiotic pretreatment; Control group 2: 10% PBS administered via gavage; Exposure to simulated high-altitude environment (6,000 m, 30 days); Fecal samples were collected and analyzed using 16S rRNA gene sequencing (relative abundance)	-	Lachnospiraceae↑ Desulfovibrionaceae↓ Desulfovibrio↓	Chen et al. (2024)
Male C57BL/6 J mice	Mice were placed in an automated animal hypoxia chamber and exposed to 5.0% oxygen (simulating a high-altitude hypoxic environment) for 48 h; fecal samples were collected and analyzed via 16S rRNA gene sequencing (relative abundance)	Desulfovibrio↑ Alloprevotella↑ Clostridium XIVa ↓	-	Li et al. (2022)
84 healthy adult Indian subjects	Subjects were divided into three distinct groups: the Leh group (high-altitude rural area) at approximately 3,500 m above sea level, the Ballabhgarh Rural group (low-altitude rural area) at approximately 228 m above sea level, and the Ballabhgarh Urban group (low-altitude urban area) at approximately 228 m above sea level; fecal samples were collected and analyzed using 16S rRNA gene sequencing	-	F/B ratio ↓ Prevotella↑ Faecalibacterium↑ Lachnospiraceae↑	Das et al. (2018)
C57BL/6 J mice	A mouse hypoxia model was established with different time points: an aerobic control group, a group exposed to hypoxia for 1 day (HD1), a group exposed to hypoxia for 3 days (HD3), and a group exposed to hypoxia for 12 days (HD12); fecal samples were collected and analyzed using 16S rRNA gene sequencing (relative abundance)	Lactobacillus↑ Bifidobacterium ↓ Akkermansia↓	Stomatobaculum ↑	Liao et al. (2025)
Adult Han Chinese males	Fecal samples were collected from the same group of subjects at multiple time points across different altitudes (243 m and 3,568 m) and exposure durations, and metagenomic sequencing was performed using the shotgun method (relative abundance)	-	Blautia A↑ Anaerostipes hadrus↑Agathobacter rectalis↑	Su et al. (2024a)
Male SD rats	SD rats were randomly divided into three groups: the plain group (390 m, 20 kPa), the moderate-altitude hypoxia group (2,800 m, 15.1 kPa), and the high-altitude hypoxia group (4,300 m, 12.4 kPa); fecal samples were collected on days 3, 7, 15, and 30 after exposure to the respective altitudes, and 16S rRNA gene sequencing was performed (relative abundance)	Akkermansia↓ Lactobacillus↑ Prevotella_9↓	Lactobacillus ↑	Bai et al. (2022)
	Participants provided fecal samples one week before entering the high-altitude region (4,500 m) and six months after arrival, and 16S rRNA gene sequencing was performed (relative abundance)	-	Blautia↑ Bifidobacterium ↑ Fusicatenibacter ↑ Anaerostipes↑ Klebsiella↓	Kan et al. (2025)
Male Wistar rats	Rats were randomly divided into two groups: a control group under normal atmospheric pressure and oxygen conditions and a low-pressure, low-oxygen model group (simulating an altitude of 5,000 m). For the first 7 days, the rats were exposed to continuous low-pressure, low-oxygen conditions; from days 8 to 28, the exposure was switched to chronic intermittent hypoxia. Fecal samples were collected and analyzed using 16S rRNA gene sequencing (relative abundance)	Prevotella↓ Lachnospira↓ Parabacteroides ↑ Alistipes ↑ Bacteroides sp. ↑	*Bacteroides ovatus* ↑ *Bacteroides fragilis*↑ Ruminococcus sp.↑ *Lactococcus garvieae* ↑ Parabacteroides ↑ Alistipes↑	Pan et al. (2022)
48 healthy women	Using deep whole-genome shotgun sequencing, the gut microbiota of 30 and 18 healthy individuals living at sea level and high altitudes, respectively, were characterized (absolute quantification)	-	*Collinsella aerofaciens* ↑ *Akkermansia muciniphila* ↑	Xiao et al. (2025)

This table summarizes the dynamic alterations of gut microbiota in humans and experimental animals during acute high-altitude exposure and long-term high-altitude acclimatization. It details the study subjects, study design, key changes in dominant microbial communities under acute exposure (with increased/decreased abundance indicated by ↑/↓. Unless otherwise specified, all comparisons are made against the baseline level at low altitude), shifts in dominant microbial taxa after long-term adaptation, and corresponding references for each included study. Note that studies labeled “(relative abundance)” did not adjust for the total microbial load, whereas studies labeled “(absolute quantification)” directly measured the absolute abundance of microbial communities. Hypoxia at high altitudes may lead to a decrease in total microbial load; thus, an increase in relative abundance could be a passive consequence of reduced populations of other microorganisms.

### 2.2 Microbial dysbiosis during the acute exposure phase

At the phylum level, the human gut microbiota is primarily dominated by Firmicutes and Bacteroidota, which together account for more than 90% of the entire relative abundance (Glazunova et al., 2025). Firmicutes is the core phylum responsible for dietary fiber fermentation and SCFAs production (Egas-Montenegro et al., 2026). The ratio of Firmicutes to Bacteroidota (F/B ratio) is often utilized as a measure of the energy metabolism ability of the gut microbiota (Cheng et al., 2022). In a study by Wang Y. et al. (2022), after 24 h of acute expose to 5,500 m altitude, the relative abundance of Firmicutes in the gut of C57BL/6 J mice decreased from 66.0 to 42.8%, while the relative abundance of Bacteroidetes increased from 13.3 to 42.8%, and the F/B ratio decreased significantly compared to the control group. The reduction of the F/B ratio has a high correlation with symptoms, including weight loss and fatigue, in individuals who have recently arrived at high altitudes (Ma X. et al., 2025). Longitudinal human studies have shown that during acute high-altitude exposure (4,500 m, 7 days), the relative abundance of SCFA-generating genera within Firmicutes, such as Faecalibacterium, Roseburia, and Blautia, has obviously decreased (Ma Y. et al., 2025). At the same time, Bifidobacterium, which is another representative producer of SCFAs, showed a continuous decrease. In human studies, the abundance of Bifidobacterium in the feces of healthy adults was significantly reduced compared to baseline levels during the initial phase of acute exposure to altitudes above 5,000 m (Kleessen et al., 2005). In mouse models, the exposure to environments that simulate an altitude of 5,500 m for 24 h was enough to cause the obvious downregulation of Bifidobacterium (Wang Y. et al., 2022). Specific changes in Bacteroidetes abundance during the acute exposure phase may have differences according to different species. For instance, experiments on rats have shown that acute exposure can increase Parabacteroides and Bacteroides, while the relative abundance of Prevotella decreases (Pan et al., 2022). In contrast, a longitudinal human study found that following acute high-altitude exposure, the relative abundance of Parabacteroides and Prevotella increased, whereas that of Bacteroides decreased (Ma X. et al., 2025). This suggests that even within the same phylum, different genera of the same species may respond to hypoxic stress in entirely different ways, and an imbalance between Bacteroides and Prevotella may act as a characteristic microbial marker of acute high altitude exposure. Bai et al. (2022) identified Firmicutes, Lactobacillus, and Akkermansia as the core microbiota in hypoxic conditions. *Akkermansia muciniphila*, the most extensively studied species within Verrucomicrobia, is localized in the intestinal mucus layer, and it uses mucin to serve as a metabolic substrate. Through adjusting the thickness of the mucus layer and promoting the expression of tight junction proteins, it occupies a unique ecological niche in maintaining the intestinal barrier (Zhai et al., 2019). During the acute exposure phase, a high-altitude exposure experiment in rats demonstrated that, when compared with the lowland control group, rats that received exposure to a 4,300-meter high-altitude environment exhibited a significant reduction in intestinal Akkermansia abundance after 7 days, and this is harmful for the stability of the intestinal mucosal microenvironment (Bai et al., 2022).

However, it is worthy to point out that although Lactobacillus is a generally acknowledged probiotic genus, research in mouse hypoxia models has discovered that acute hypoxia exposure leads to a sharp increase in the relative abundance of Lactobacillus in the gastric and small intestinal microbiota, where it gains absolute dominance. Its rapid acid-producing features are regarded as an important factor that causes early decreases of microbial diversity and the inhibition of other genera (such as Bifidobacterium), reflecting the acute stress response and reorganization of the microbiota in hypoxic environments (Liao et al., 2025). It should be noted that in the acute hypoxia stage, the phenomenon of observing lactobacillus in the upper digestive tract may be strain-specific: the lactobacillus genus contains a large number of complex species. Some strains, which may have rapid acid-producing ability and strong adaptability, show a strong ability to occupy ecological niches at the early stage of stress; however, not all strains have consistent ecological effects. As the exposure time increases and the host physiological state adapts, the abundance of lactobacillus decreases to be close to the aerobic level, and then the microbial diversity recovers, which indicates that this short-term advantage is part of the dynamic adaptation process (Liao et al., 2025). Meanwhile, certain Lactobacillus strains with anti-inflammatory and barrier-protective functions (such as *Lactobacillus johnsonii* YH1136, isolated from the feces of healthy Tibetans) may naturally become dominant in high-altitude environments, thus achieving the functional transition from stress biomarkers to adaptive protective bacteria (Wan et al., 2022). Furthermore, Ruminococcus and Oscillibacter within the phylum Firmicutes also have the tendency to increase during acute high-altitude exposure (Ma Y. et al., 2025). Given that *Ruminococcus gnavus* produces pro-inflammatory polysaccharides that activate the toll-like receptor 4 (TLR4) pathway (Henke et al., 2019), and that Oscillibacter is significantly positively correlated with the expression levels of pro-inflammatory cytokines such as interleukin-6 (IL-6), interleukin-*β* (IL-1β), and tumor necrosis factor-*α* (TNF-α) in intestinal tissues (Tong et al., 2021), the enrichment of these two genera may together make contribution to the potential inflammatory condition of the intestine observed during the early stages of high-altitude exposure.

### 2.3 Microbiome remodeling following high-altitude acclimatization

Longitudinal studies have indicated that during the adaptation process following migration to high altitudes, the structure of the gut microbiota undergoes dynamic changes, gathering the features of high-altitude resident populations (Jia et al., 2020). Although the F/B ratio in low-altitude populations drops sharply during the acute exposure phase due to the depletion of the Firmicutes phylum caused by hypoxic stress, this ratio partially recovers as the host initiates an adaptive response (Ma X. et al., 2025). However, the long-term adaptation process is accompanied by a phylum-level reshaping of the gut microbiota, which is expressed as an increase in Bacteroidetes and a decrease in Firmicutes, that is, a decrease in the F/B ratio (Jia et al., 2020; Han et al., 2024). Studies have indicated that even though the F/B ratio in populations living long-term at high altitudes is obviously lower than that in low-altitude populations, these people have increased the amount of bacterial groups with special metabolism functions to optimize their adaptation to energy metabolism (Jia et al., 2020). For example, Prevotella, which is enriched in the Tibetan population, can effectively degrade complex dietary polysaccharides. Meanwhile, several genera associated with SCFAs production, such as Butyricimonas and Roseburia, also exhibit characteristic changes during adaptation to the plateau. These function-specialized bacterial communities provide a more refined energy metabolism optimization strategy, independent of changes in the overall F/B ratio, through increasing dietary fiber fermentation and producing SCFAs available to the host, hence solving the energy challenges of the hypoxic high-altitude environment (Jia et al., 2020). Meanwhile, researchers point out that among the Tibetan population, as indigenous residents of the plateau, the obvious enrichment of Prevotella in their gut microbiota is associated with a lower risk of cardiovascular disease (Lv et al., 2023; Ma et al., 2021a). Bacteroides is enriched in populations migrating at low altitudes. Early work by Adak et al. (2013) has discovered that when 15 days of acclimatization at an altitude of 3,505 m are finished, the abundance or number of cultivable Bacteroidetes, especially Bacteroides, in human feces has an obvious increase compared with initial baseline levels. A study by Ma X. et al. (2025) revealed, from an ecological community perspective, 127 healthy Han Chinese volunteers showed that as altitude increases, a greater proportion of individuals, compared to the baseline, shift their gut microbiota to a Bacteroides-dominant BA enterotype following exposure to mid-altitude environments. Comparative analyses have shown that even if people live in the same high-altitude area, the gut microbiota of indigenous Tibetan populations have the feature of relative enrichment of Prevotella, whereas long-term Han Chinese migrants tend to exhibit greater enrichment of Bacteroides (Li J. et al., 2016; Ma et al., 2021b). It should be noted that the differences in the gut microbiota between the Tibetan and Han populations may be influenced by both the genetic background and the long-term traditional dietary patterns. Each factor may independently explain some of the observed microbial characteristics. The Tibetan highland population represents a unique evolutionary lineage shaped by thousands of years of hypoxic selection; compared with the lowland Han people, they carry specific adaptive variations in two hypoxia-related genes, namely, the protein with an endothelial PAS domain 1 (EPAS1) and Egl-9 family HIF 1 (EGLN1), which fundamentally changes the hypoxic perception, vascular reactivity, and basal metabolic rate (Peng et al., 2017; Fan et al., 2026), and may promote the formation of the gut microbiota characterized by the enrichment of Prevotella and a low F/B ratio in the Tibetan population. Meanwhile, gut microbiota composition, especially the categorization of enterotypes, is closely related to the host’s long-term dietary patterns. Studies have indicated that Bacteroides-dominant enterotypes are typically connected with Western diet modes that have high content of animal protein and saturated fat, whereas Prevotella-dominant enterotypes are connected with diet mode that has high content of carbohydrate (Wu et al., 2011). This reflects the differences in the traditional diet structures of the two ethnic groups: the Tibetan diet typically includes more high-fiber grains, whereas Han Chinese immigrants may keep more features of an urban diet, which is led by refined carbohydrates and animal-based foods (Li K. et al., 2016). However, longitudinal studies under the condition of strictly controlling diet and lifestyle variables, have shown that after Han Chinese migrate to high-altitude places and reside there for 6 months, there is an obvious rise in the abundance of Prevotella, especially *Prevotella copri*, which demonstrates that its microbial composition partially approaches the Tibetan baseline (Han et al., 2024). But then, under different genetic backgrounds and dietary habits, exposure to low oxygen alone may not be sufficient to overcome population-level differences.

The phylum Proteobacteria, as one category inside the gut microbiota, is relatively low in abundance but of significant functional importance. It contains many Gram-negative bacteria capable of synthesizing LPS, among which several genera of the Enterobacteriaceae family are representative of opportunistic pathogens (Yin et al., 2025). After long-term adaptation, the relative abundance of Proteobacteria generally displays an obvious decreasing tendency in plateau populations, including Han Chinese migrants and indigenous Tibetans, which forms a huge contrast with that in Han Chinese populations living at low altitudes (Zhao et al., 2024). Among Han Chinese populations living long-term at low altitudes, *Escherichia coli* has been identified as a significantly enriched opportunistic pathogen, while its content is lower in native Tibetan populations living on the plateau (Han et al., 2024), which may help reduce the risk of infections that are caused by specific opportunistic pathogens among plateau populations.

## 3 Pathogenic mechanisms of cardiovascular disease induced by gut microbiota dysbiosis at high altitudes

### 3.1 The pathogenic role of gut microbiota-mediated metabolic dysregulation in cardiovascular disease

**Figure 1:**
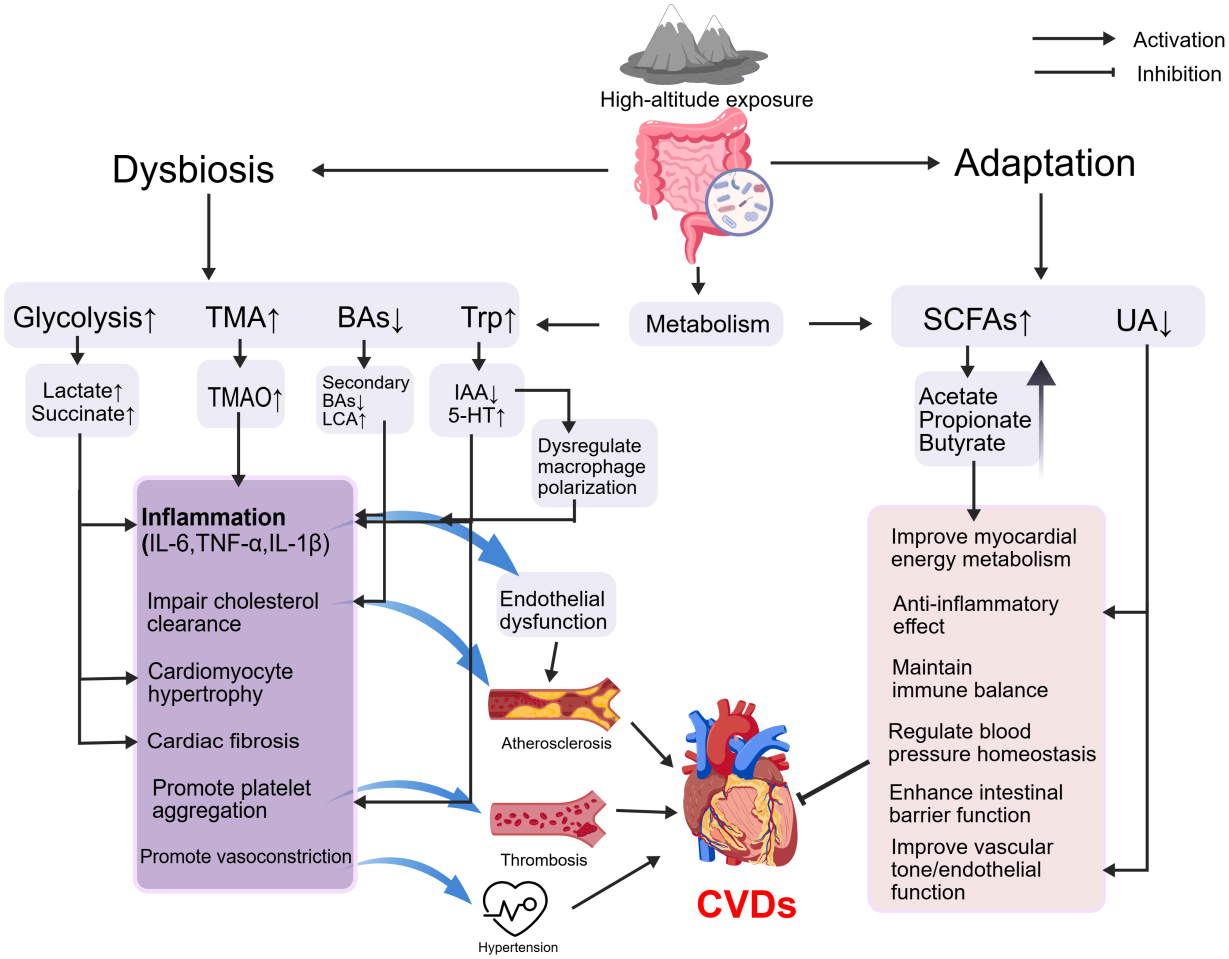
Dual effects of gut microbiota and its metabolites on cardiovascular diseases under high-altitude exposure. Acute exposure-induced gut dysbiosis leads to metabolic disturbances that promote cardiovascular disease development. In contrast, adaptive remodeling of the gut microbiota produces beneficial metabolites that exert protective effects against cardiovascular damage. Created with BioGDP.com.

The structure and function remodeling of the gut microbiota induced by high-altitude environments mainly contributes to the pathogenesis of cardiovascular disease by mediating destruction in the host’s metabolic networks. The gut microbiota is considered a metabolic organ of the human body; changes in the types and concentrations of its metabolites (e.g., SCFAs, energy metabolism intermediates, TMAO, and bile acids) directly adjust the physiological functions of the host’s cardiovascular system. These metabolites act on target cells like cardiomyocytes and vascular endothelial cells through the bloodstream, forming multi-dimensional pathogenic paths through adjusting energy supply, inducing inflammatory responses, or disrupting lipid metabolism (Figure 1).

#### 3.1.1 Pathogenicity of energy metabolic dysregulation

High-altitude environments cause host energy metabolism imbalances and abnormal accumulation of energy metabolism intermediates through pushing gut microbiota reorganisation. In high-altitude migrants with abnormal cardiac health, the abundance of strains such as *Veillonella rogosae* and Streptococcus rubneri, which have a positive correlation with aerobic metabolism pathways, is greatly decreased. These strains keep energy metabolic balance through joining the malate–aspartate shuttle, tricarboxylic acid cycle, and oxidative phosphorylation pathway. The reduction in their abundance is closely associated with the inhibition of mitochondrial electron transport chain function, leading to a raised proportion of energy obtained from glycolysis, hence causing serum lactate accumulation (Zhou et al., 2025). Concurrently, the obvious increase in Lactobacillus abundance during acute exposure, together with its rapid acid-producing characteristics, exacerbates this phenomenon (Table 1). Lactate has the function of a signal molecule, which can activate the specific receptor GPR81 (succinate receptor, one of G protein-coupled receptors (GPCRs)), thus influencing the inflammation of blood vessels and their function. More importantly, lactate-driven lactic acid modification of histones and non-histones plays a central role in inflammatory repair, fibrotic remodeling, as well as the onset and development of heart failure following myocardial infarction (Song K. et al., 2025). In atherosclerosis, lactate activates the NADH/CtBP1-dependent pathway through endothelial cell MCT1, removing FOXP1 transcriptional repression of adhesion molecule genes, thereby driving vessel inflammation in atherosclerosis (Li P. et al., 2025). Animal studies have confirmed that under hypoxic conditions, gut microbiota-mediated enhancement of glycolysis and lactate accumulation causes the rise of basal and compensatory glycolysis speed in cardiomyocytes, exacerbating myocardial injury and cardiac hypertrophy. With supplementation of the aforementioned bacterial strains, *Veillonella rogosae* and Streptococcus rubneri, or their metabolites, can significantly inhibit this process and improve myocardial energy metabolism (Zhou et al., 2025). In addition, study that is carried out with high-altitude mammal models (such as yaks) indicates that the metabolic reprogramming of gastrointestinal epithelial cells and the cell-type specific colonization of Bacillus will result in the accumulation of key metabolites like succinate and lactate in epithelial cells (Huang C. et al., 2026); research on people living in high-altitude areas also shows that there is an increase in the colonization of *Clostridium symbiosum*, a gut bacterium that produces succinate, promoting the accumulation of gut-derived succinate (Zhou et al., 2026). In the human umbilical vein endothelial cell (HUVEC) model, the accumulation of succinate causes the mitochondrial damage of endothelial cells, which then leads to the pyroptosis of cells and promotes the pathological process of atherosclerosis (Huang et al., 2025). Further research in rodents shows that succinate can directly trigger pathological cardiomyocyte hypertrophy by activating the GPR91 on cardiomyocytes (Aguiar et al., 2014); the GPR91 is also expressed in human heart tissue and the activation of the GPR91-phosphoinositide 3-kinase/protein kinase B (PI3K/Akt) pathway is detected in human right ventricular hypertrophy (RVH) tissue samples (Yang et al., 2016). Therefore, we conjecture that the accumulation of succinate in the intestines at high altitudes may also play a part in the occurrence and development of cardiovascular diseases as well.

#### 3.1.2 Pathogenicity of TMAO

In high-altitude environments, the interaction between gut microbiota and diet can greatly raise the activity of the trimethylamine (TMA)-TMAO metabolic pathway, making it a key mediator of cardiovascular disease. Recent nutritional epidemiological studies have pointed out that people who live in high-altitude areas eat relatively higher proportions of high-choline and high-carnitine foods, such as meat and dairy products, in their diets (Huang Y. et al., 2026). Representative strains of choline TMA-cleaving enzyme (cutC)-rich bacterial genera in the gut microbiota, such as Lachnoclostridium and Clostridium, are able to change eating source precursor substances like choline and L-carnitine into TMA, which, after it goes into the liver through the portal vein, is oxidized by flavin monooxygenase 3 (FMO3) to form TMAO. The abundance of these bacterial genera in patients with atherosclerosis is much higher than that in healthy individuals (Cai et al., 2022). An animal experimental study conducted in a high-altitude region (4,500 m above sea level) further revealed that a high-fat diet can simultaneously upregulate the expression of hepatic FMO3 and significantly increase plasma TMAO levels (Sang et al., 2021). Meanwhile, evidence from animal studies also indicates that high-altitude hypoxia can cause an increase in the expression of hypoxia-inducible factor-1α (HIF-1α) in the liver, directly binding to the FMO3 promoter to promote its transcription, and upregulating the TMA/FMO3/TMAO pathway, thus leading to an elevation in the level of TMAO in the plasma, which is positively correlated with the development of gallbladder cholesterol stones (GCS) (Luo M. et al., 2024). Clinical data show that the plasma TMAO levels of gallstone patients are significantly higher than those of people without stones. Furthermore, in high-altitude female populations, the increased levels of total cholesterol and apolipoprotein B are associated with the development of GCS, which suggests that dyslipidemia in a hypoxic environment may play a part in the formation of GCS through the HIF-1α/TMAO axis (Chen et al., 2019; Luo Z. et al., 2024).

Elevated plasma TMAO levels promote the onset and progression of cardiovascular disease through multifaceted pathophysiological mechanisms. Regarding lipid metabolism dysregulation, clinical and epidemiological evidence has established a strong association between TMAO and atherosclerosis risk in humans (Koeth et al., 2013). Preclinical studies further elucidating the underlying mechanisms have demonstrated that TMAO reduces the efflux and clearance of cholesterol from peripheral tissues by inhibiting reverse cholesterol transport and promotes the uptake of modified lipoproteins by macrophages, thereby accelerating the formation of foam cells in the mouse model (Koeth et al., 2013). Concurrently, preclinical *in vitro* studies have demonstrated that TMAO can exacerbate intracellular oxidative stress and inhibit cholesterol efflux by suppressing the Nrf2/ABCA1 pathway in macrophages, thereby directly promoting lipid accumulation in foam cells (Luo M. et al., 2024). These effects collectively lead to abnormal lipid deposition in the vascular wall, thereby driving the development of atherosclerotic plaques. Regarding vascular inflammation, studies utilizing primary human aortic endothelial cells (HAECs) and vascular smooth muscle cells have demonstrated that, even physiological concentrations of TMAO can activate the mitogen-activated protein kinase (MAPK) and nuclear factor κB (NF-κB) signaling pathways in vascular endothelial cells and smooth muscle cells, hence it significantly raises the expression level of various inflammatory factors and adhesion molecules, which include IL-6, intercellular adhesion molecule-1 (ICAM-1), and E-selectin, directly promoting the recruitment and adhesion of leukocytes to the vascular endothelium (Seldin et al., 2016). Chen et al. found in HUVECs and ApoE−/−mice that TMAO inhibits the SIRT3-SOD2 axis, leading to excessive accumulation of mitochondrial reactive oxygen species (ROS) and activation of nod-like receptor protein 3 (NLRP3) inflammasomes, thereby promoting the maturation and release of pro-inflammatory factors (e.g., IL-1*β*), while upregulating the expression of ICAM-1 and matrix metalloproteinase-9 (MMP-9), therefore it exacerbates local vascular inflammatory responses and accelerates the progression of atherosclerosis lesions (Chen et al., 2017). In the aspect of thrombogenesis, in vitro experiments on human platelets have demonstrated that TMAO enhances platelet responsiveness to agonists, such as collagen, thrombin, and ADP, to promote platelet aggregation (Zhu et al., 2016); meanwhile, clinical cohort studies show that the rise of plasma TMAO levels in participants are independentlyrelated to the increased risk of thrombotic occurrences (myocardial infarction or stroke) within 3 years. Animal models and cellular experiments further suggest that TMAO can also specifically induce the expression of tissue factor (TF) in vascular endothelial cells, significantly strengthening thrombogenesis after arterial injury (Witkowski et al., 2022). In the process of myocardial remodeling and heart failure occurrence, animal experimental evidence indicates that TMAO, through activating the TGF-β1/Smad3 signaling pathway, directly induces cardiomyocyte hypertrophy, manifested as increased cell area and upregulation of fetal gene expression, such as ANP and β-MHC. At the same time, TMAO promotes myocardial stromal fibrosis by promoting the activation of cardiac fibroblasts and excessive synthesis and deposition of collagen (mainly types I and III) (Li A. et al., 2019). Although clinical observations show that the circulating TMAO levels in patients with heart failure are significantly increased and are related to the severity of the disease and the poor prognosis (Ding et al., 2025), however, further human tissue studies or precise intervention trials need to be conducted to verify whether TMAO directly mediates myocardial hypertrophy and fibrosis in humans through the above-mentioned pathways. Furthermore, in the mouse model, TMAO has the ability to elevate blood pressure by activating the PERK/ROS/CaMKII/PLCβ3 signaling axis in vascular smooth muscle cells, therefore magnifying angiotensin II (Ang II)-induced intracellular calcium release and vasoconstriction (Jiang et al., 2021). However, there is currently a lack of interventional studies in high-altitude populations, and the specific function of TMAO and its relevant pathways in the progression of cardiovascular diseases in these populations still requires further investigation.

#### 3.1.3 Pathogenicity of bile acid metabolic disorders

The gut microbiota is a core regulator of bile acid metabolism. Dysbiosis in high-altitude environments disrupts the dynamic balance of bile acid synthesis, transformation, and reabsorption, hence it mediates the damage of cardiovascular damage. Multiple animal experiments and human studies have indicated that exposure to high altitude or hypobaric hypoxia can alter the composition of the gut microbiota, accompanied by disturbances in secondary bile acid metabolism. In a rat model subjected to prolonged hypobaric hypoxia, the abundance of bacterial genera with bile salt hydrolase (BSH) activity, like Bacteroides and Alistipes, significantly increased. This was accompanied by a continuous decrease in the relative abundance of the secondary bile acid lithocholic acid (LCA) in the feces. Meanwhile, in rat plasma, the relative abundance of bile acids with cardioprotective effects, such as cholic acid (CA), chenodeoxycholic acid (CDCA), deoxycholic acid (DCA), and glycoursodeoxycholic acid (GUDCA), had an obvious decrease, whereas the levels of LCA, which may be cardiotoxic, had a significant increase (Pan et al., 2022). Similarly, in Gansu pygmy mice, which are naturally adapted to hypoxia, hypoxic treatment caused a rise in the abundance of microbial communities (such as Lactobacillus, Bacteroides, and Bifidobacterium) that have ability to secrete BSH and take part in bile acid deconjugation and metabolism, and also resulted in a significant decrease in LCA levels in colonic contents (Yang L. et al., 2025). In human studies, the gut microbiota enterotypes (Bacteroides-dominated) associated with altitude exposure were also connected with downregulated levels of various secondary bile acids in serum, such as DCA, glycodeoxycholic acid (GDCA), ursodeoxycholic acid (UDCA), and GUDCA (Ma X. et al., 2025). Dysbiosis of the gut microbiota dynamically regulates the activation state of the farnesoid X receptor (FXR) by altering the composition, metabolic transformation, and pool size of bile acids. When the FXR pathway is inhibited or functionally impaired due to changes in the bile acid profile, it on one hand relieves the inhibition of cholesterol 7α-hydroxylase (CYP7A1) (Trauner and Fuchs, 2022) but also impairs cholesterol reverse transport, leading to impaired systemic cholesterol clearance and hypercholesterolemia (Chiang et al., 2020). Concurrently, inhibited FXR activation disrupts hepatic lipid metabolism, promotes triglyceride synthesis, and results in a lipoprotein profile that promotes atherosclerosis. Furthermore, dysregulation of bile acid signaling (including the FXR and Takeda G protein-coupled receptor 5 (TGR5) pathways) can promote inflammatory responses in the vascular wall (Chiang et al., 2020). These metabolic and inflammatory dysregulations collectively increase the risk of atherosclerotic cardiovascular disease (ACVD). At the local vascular level, secondary bile acids can act directly on the cardiovascular system as signaling molecules. The accumulation of highly hydrophobic LCA may exert direct cytotoxic effects on vascular endothelial cells (Kundu et al., 2017); while a deficiency in hydrophilic, protective bile acids (such as UDCA) weakens their cardiovascular protective effects, which are mediated through anti-inflammatory, antioxidant, and receptor-activating pathways (e.g., TGR5), preventing them from effectively suppressing inflammatory responses in the vascular endothelium (such as downregulating NF-κB) and oxidative stress, thereby collectively promoting the progression of cardiovascular pathologies such as atherosclerosis (Mihajlović et al., 2024; Punzo et al., 2024).

#### 3.1.4 Pathogenicity associated with tryptophan metabolites

The indole pathway is one of the main routes of tryptophan metabolism, in which the gut microbiota plays a critical role by generating various indole derivatives (Lu et al., 2025). On account of differences in their enzyme systems, different gut bacterial genera metabolize tryptophan into distinct indole derivatives. For example, numerous bacteria carrying tryptophanase, such as *Escherichia coli*, can break down tryptophan into indole (Sinha et al., 2024). Bacteroides and others generate indole-3-acetic acid (IAA) through a series of consecutive enzymatic reactions (Jia et al., 2024), while Clostridia, represented by *Clostridium sporogenes*, can change tryptophan into compounds that include indolepropionic acid (IPA) and indole lactic acid (ILA) through pathways such as Stickland fermentation (Sinha et al., 2024). Metabolomic analysis of human plasma shows that IAA levels are significantly downregulated during acute high-altitude hypoxia exposure (Gao et al., 2023). IAA alleviates atherosclerosis by inhibiting the TLR4/MyD88/NF-κB pathway and promoting M2 macrophage polarization. The decrease in IAA levels leads to an imbalance in M1/M2 macrophage polarization, with pro-inflammatory M1 macrophages becoming dominant, thereby accelerating vascular wall inflammation and plaque instability (Liu et al., 2025b). In addition, studies have shown that IAA, through activating the deubiquitinating enzyme USP40, can stabilize the HSP90β protein, thereby maintaining the integrity of the pulmonary microvascular endothelial barrier (Shaheen et al., 2024). The lack of IAA leads to the disruption of endothelial cell junctions, the rise of vascular permeability, and the promotion of atherosclerosis and myocardial injury. However, quantitative data specifically targeting plasma indole derivatives in high-altitude organisms, especially humans, remain scarce; most studies rely on predictions based on microbial function or extrapolations from animal models. Studies have demonstrated that transferring gut microbiota from high-altitude animals to low-altitude SD rats through fecal microbiota transplantation (FMT) can significantly reshape the gut microbiota structure of the recipient rats, significantly increase the production of anti-inflammatory metabolites such as indole-3-lactic acid (ILA), effectively reduce pulmonary artery pressure, alleviate right ventricular hypertrophy, and relieve hypoxic pulmonary hypertension (Chen Z. et al., 2025). This may be an adaptive protecting mechanism that high-altitude living organisms have formed under the effect of long-term hypoxic selective pressure, which is completed through reshaping the metabolic capacity of the gut microbiota for indole derivatives.

During the high-altitude adaptation phase, analysis of gut microbiota function in Han Chinese individuals who have resided at high altitudes for 6 months has revealed significant enrichment of the tryptophan biosynthesis pathway (Han et al., 2024). Tryptophan is a precursor of the neurotransmitter serotonin (or 5-hydroxytryptamine(5-HT)). The microbiota-derived SCFAs promotes the 5-HT generation through increasing the mRNA and protein expression level of tryptophan hydroxylase 1 (TPH1), which is the rate-limiting enzyme for intestinal serotonin synthesis (Reigstad et al., 2014; Liu K. et al., 2021). Studies have revealed that in the mouse model, microbiota-induced increases in intestinal 5-HT not only affect gastrointestinal motility but also are absorbed by platelets through the SERT transporter, enhancing platelet aggregation and activation, therefore probably affecting the risk of thrombosis (Yano et al., 2015). Patients with inflammatory bowel disease (IBD) exhibit EC proliferation and significantly elevated intestinal 5-HT levels, making them a high-risk group for venous thromboembolism (VTE) (Shajib et al., 2017; Uh et al., 2025), which may be associated with excessive 5-HT release induced by intestinal inflammation and enhanced platelet activation. The hypoxia-induced barrier stress differs from the intestinal barrier characterized by infiltrative inflammation triggered by chronic ulcers in IBD patients; however, it might also cause the level of intestinal 5-HT to increase: intestinal endothelial cells function as oxygen sensors, and hypoxia activates TPH1 through the HIF-1*α* pathway to promote the synthesis and secretion of 5-HT (Haugen et al., 2012). Nevertheless, a human cross-sectional study discovered that after being in a high altitude for a long time, the level of peripheral plasma 5-HT decreased (Zhong et al., 2021). We suggest that this decrease in peripheral serotonin might be associated with central consumption or increased peripheral metabolism, rather than the decrease in intestinal 5-HT; at the same time, the researchers noted that the study did not exclude the influence of confounding variables such as lifestyle and diet. We did not find any direct research regarding the relationship between the high-altitude environment and human serotonin levels as well as thrombus risk, nor did we have studies that compare the serotonin levels in the blood and intestines of residents in high-altitude areas and low-altitude areas. Therefore, the aforementioned pathway that the increase in intestinal 5-HT levels in high-altitude environments will increase thrombus risk is only based on model conclusions and inferred from similar clinical scenarios. Future longitudinal studies are needed to simultaneously measure the 5-HT and platelet activation markers in the fecal or peripheral blood samples collected under high-altitude environments. In order to figure out the extent to which the gut microbiota, the SCFAs produced by them, and the hypoxic stress will affect the serum serotonin level and the risk of thrombus formation in the high-altitude environment.

### 3.2 Inflammation and immune activation

Inflammation is one core danger factor for cardiovascular disease (Wu and Chiou, 2021). High-altitude environments induce gut microbiota dysbiosis, therefore causing systemic inflammatory and immune responses. The core mechanisms that underlie this process are closely connected with the breaking of the intestinal barrier, the release of inflammatory mediators, and the abnormal function of immune cells, as confirmed by numerous clinical and experimental studies.

#### 3.2.1 Disruption of intestinal mucosal lymphoid tissue and impairment of the intestinal barrier

The gut-associated lymphoid tissue (GALT), which consists of Peyer’s patches, isolated lymphoid follicles, and intraepithelial lymphocytes, acts as an anatomical basis for the gut immune response. The integrity of these structures and the immune homeostasis maintained with the gut microbiota are crucial for the normal functioning of GALT (Hooper and Macpherson, 2010). Acute exposure to high-altitude hypoxia directly disrupts the intestinal mucosal barrier and the functional homeostasis of GALT. Research indicates that within Peyer’s patches, a key component of GALT, rats exposed to a simulated hypobaric hypoxia environment at 7,620 meters, there are obvious changes in the composition of immune cells. The manifestation is that the initial number of T cells is significantly reduced, and the proportion of innate immune cell populations, such as natural killer cells and dendritic cells, becomes higher. This shows that hypoxic stress has reshaped the pattern of intestinal immune responses (Khanna et al., 2019). Meanwhile, acute high-altitude hypoxia can directly damage the physical barrier function of the intestinal mucosa, which is manifested by the down-regulation or abnormal distribution of tight junction proteins, such as occludin, claudin-1, and ZO-1, as well as the reduction of goblet cells and the thinning of the mucus layer (Liu et al., 2024; Qi et al., 2025). In addition, the dysbiosis of the gut microbiota and the alteration of its metabolites, such as the decrease of SCFAs, will further cause problems with the barrier function (Liu et al., 2024). It is worth noting that beneficial bacteria like Bifidobacterium and Akkermansia have been confirmed to be able to promote the secretion of mucus and maintain the integrity of the mucus layer (Gutierrez et al., 2023; Mo et al., 2024); if their abundance decreases under acute hypoxic conditions, it may further intensify the process of mucus barrier damage (Table 1). Moreover, in high-altitude areas, the phospholipid metabolites that originate from the increase of Desulfovibrio and are like phosphatidylethanolamine and phosphatidylcholine are presented to γδ T cells through the CD1d molecules on the surface of intestinal epithelial cells, thus resulting in the activation of the latter and then the massive secretion of IL-17A (Li et al., 2022). IL-17A causes the local inflammatory responses to persist, disrupts the intestinal epithelial barrier, and worsens the hypoxia-induced intestinal injury.

A direct consequence of the impairment of the intestinal barrier is the invasion of antigens. After male SD rats are exposed to hypobaric hypoxia for 21 days leads to a significant increase in serum levels of zonulin, which is a marker of intestinal permeability, increases (Bakshi and Mishra, 2025). The upregulation of zonulin and the downregulation of tight junction proteins are closely associated with the adhesion and invasion of pathogenic bacteria, and serum LPS levels are significantly elevated in affected individuals (Ciccia et al., 2017). Zonulin is a physiological regulator that regulates intestinal permeability. A meta-analysis about cardiovascular diseases shows that in the serum or plasma of patients with cardiovascular diseases like coronary heart disease, the levels of zolin protein and lipopolysaccharide are obviously higher than those of healthy people (Xiao et al., 2024). Antigens that enter the circulatory system (like components of bacterial cell walls and incompletely metabolized metabolites) can directly activate systemic immune cells via the circulatory system, triggering an inflammatory response (Robles-Vera et al., 2025). In rats that are exposed to high altitude, the supplementation of Bifidobacterium probiotics can enhance the integrity of the intestinal barrier, decrease the levels of inflammatory cytokines in the serum, and also ease myocardial hypertrophy (Hu et al., 2022). On the contrary, disruption of GALT and mucosal function weakens the regulation over the gut microbiota, enabling pathogenic bacteria to multiply in large numbers and thus creating a vicious cycle.

#### 3.2.2 HIF-1*α* overexpression-mediated inflammatory immune responses

The overexpression of HIF-1α because of high-altitude hypoxia not only plays a vital role in hypoxia adaptation but also seems to be a central hub connecting the dysbiosis of gut microbiota, the damaged barrier, and systemic inflammation. A hypobaric hypoxic environment will cause a significant rise in the expression of HIF-1α in the intestinal mucosal tissue, and the expression level gradually increases as the altitude goes up (Zhang et al., 2015). Under normal conditions, physiological hypoxia will enhance the intestinal barrier function by activating the HIF-1α signaling pathway; this effect is partially dependent on the upregulation of barrier-related genes such as tight junction protein claudin-1 and mucin 2 (MUC2) (Wu et al., 2025). However, an in-vivo study in healthy subjects shows that after a rapid passive ascent to 3,830 meters, the peripheral oxygen saturation (SpO₂) decreases significantly, being 11% lower than the baseline at 24 h and still below the baseline at 72 h; the HIF-1α mRNA level presents a parabolic change, increasing by 59% compared with the baseline at 24 h and reaching the peak, then returning to the baseline level at 72 h, which implies that in the sub-acute phase of 24 h, the early stress mediated by HIF-1α plays a leading role (Malacrida et al., 2019). When the organism is acutely exposed to high-altitude environments, the expression level of HIF-1α exceeds physiological regulatory limits, potentially shifting its predominant protective role: it is experimentally shown that under the condition of simulating an altitude of 4,767 m, the expressions of HIF-1α protein and inducible nitric oxide synthase (iNOS) protein will be simultaneously obviously upregulated, and the two are positively correlated as well (Zhang et al., 2015). This indicates that HIF-1α may destroy the integrity of the intestinal epithelial barrier by activating iNOS, thus resulting in redox imbalance and ultimately causing pathological changes, such as intestinal mucosal villus atrophy and shedding (Zhang et al., 2015; Yu et al., 2026). Meanwhile, under the condition of high-altitude hypoxia, HIF-1α expression level in Peyer’s patches is positively correlated with the apoptosis rate of T cells and negatively correlated with the number of CD3 + T cells (Zhang et al., 2016). This suggests that the overexpression of HIF-1α may directly damage the function of the lymphocyte pool and GALT function by inducing T-cell apoptosis. The pathogenic bacterium Desulfovibrio possesses the ability to reduce sulfate, and its accumulation can bring about more serious inflammatory responses (Liao et al., 2023). Hydrogen sulfide (H₂S) produced by Desulfovibrio can stabilize HIF-1α by inhibiting prolyl hydroxylase (Munteanu, 2023). Therefore, we hypothesize that the enrichment of Desulfovibrio in high-altitude regions may further activate the HIF-1α pathway by generating H₂S, thus strengthening inflammatory damage.

HIF-1α is directly bound to the hypoxia response elements of the pro-inflammatory cytokine genes, and then the transcription of them is promoted correspondingly (Lampiasi and Russo, 2026). In the intestinal tissues of mice exposed to high-altitude hypoxia, the significant upregulation of HIF-1α protein expression can be observed, and at the same time, the mRNA and protein levels of pro-inflammatory cytokines like IL-6 and TNF-α. The effective downregulation of the expression of these factors and the reduction of the damage to the intestinal mucosal barrier can be attained by inhibiting the HIF-1α/glycolysis pathway (Yan et al., 2024). Additionally, HIF-1α, which plays a crucial role in the metabolic reprogramming related to cardiovascular diseases, also has its expression increased during the early stage of myocardial hypertrophy and in heart failure (Sant’Ana et al., 2023).

#### 3.2.3 Pathogenic bacteria and LPS-mediated systemic inflammation

The hypoxic environment in high-altitude areas can disrupt the integrity of the intestinal mucosal barrier and also lead to the imbalance of the gut microbiota, which presents as a decrease in beneficial bacteria and the excessive growth of potential pathogenic bacteria. Under such circumstances, the increased intestinal permeability will allow endotoxins (LPS) and intestinal bacteria to transfer into the systemic circulation. These transferred LPS, as powerful inflammation activators, stimulate the body to produce a large number of pro-inflammatory cytokines, thereby triggering the systemic inflammatory cascade reaction. This is closely related to the systemic inflammatory state that is observed following high-altitude exposure (He et al., 2018). In animal models, commodity pigs raised in high-altitude areas have a significantly higher concentration of LPS in their serum compared with the low-altitude control groups, accompanied by upregulation of inflammatory factors like IL-1β and IL-6, as well as the situation of intestinal barrier damage (Luo et al., 2022). The release of LPS synergizes with the destruction of intestinal mucosal lymphoid tissue and barrier damage. The impaired function of GALT leads to the decline of the mucosal immune surveillance, making it easier for pathogenic bacteria and their products to remain and pass through the barrier; meanwhile, the increased permeability of the intestinal epithelial barrier will accelerate the entry of microbial products such as LPS into the bloodstream (Hofer and Speck, 2009). LPS can also act as a persistent source of inflammatory stimulation. The integrity of the barrier is further disrupted by activating the TLR4 signaling pathway in intestinal epithelial cells, leading to more severe barrier damage and facilitating the invasion of pathogenic bacteria (Guo et al., 2013). Furthermore, LPS can upregulate HIF-1α expression through pathways such as TLR4/NF-κB (Zhao et al., 2022), and the accumulation of HIF-1α in turn provides positive feedback to enhance the activity of the TLR4 signaling pathway, thus amplifying the cellular response to inflammatory stimuli (Yao et al., 2013). This positive feedback loop continuously drives local inflammatory responses, promoting the release of pro-inflammatory cytokines IL-1β, TNF-α, IL-6, and M1 macrophage polarization (Guo et al., 2023), and may exacerbate endothelial dysfunction and a pro-coagulant state, jointly contributing to the instability of atherosclerotic plaques and an increased risk of thrombus formation. However, direct human proof that the positive feedback loop is the rate-limiting step for altitude-associated atherogenesis is still lacking.

Once LPS enters the circulatory system, it activates immune cells such as macrophages through the TLR4/NF-κB/ROS pathway, resulting in the release of a large number of pro-inflammatory factors (Ohtsu et al., 2017). Animal studies have confirmed that in ApoE⁻/⁻ mice, LPS stimulation can significantly aggravate the atherosclerotic lesions of the aorta and also increase the levels of autoantibodies and inflammatory factors in the serum; such an effect can be mitigated by the TLR4 inhibitor TAK-242, which clearly defines the pathogenic role of the LPS/TLR4 pathway in atherosclerosis (Ni et al., 2013). Clinical studies have confirmed that the systemic inflammatory response caused by acute high-altitude exposure is characterized not only by a significant increase in pro-inflammatory factors in the serum of exposed individuals but also by a decrease in anti-inflammatory factors like IL-10, thus disrupting the pro-inflammatory/anti-inflammatory balance (Ullah et al., 2025). TNF-α and IL-1*β* are key mediators of vascular endothelial dysfunction. They can stimulate vascular endothelial cells, upregulating the expression of adhesion molecules VCAM-1 and ICAM-1 on their surface by activating inflammatory signaling pathways such as nuclear factor κB (NF-κB) within endothelial cells. This causes the adhesion and invasion of mononuclear cells in the cycle into the vascular wall, which is the initial stage of the development of atherosclerosis (Kopych et al., 2025). At the same time, TNF-α can directly reduce the synthesis of nitric oxide (NO) that has vasodilatory, anti-inflammatory, and antithrombotic functions by down-regulating the expression and activity of endothelial nitric oxide synthase (eNOS), while promoting the generation of ROS, thus further aggravating oxidative stress and cell damage (Kopych et al., 2025). In the process of atherosclerosis, inflammatory mediators can also trigger the coagulation cascade reaction by inducing the expression of tissue factor (TF), and can also promote platelet activation and inflammatory amplification through pathways like the activation of protease-activated receptors (PARs) (Neumann et al., 1997; Demetz and Ott, 2012); meanwhile, they can upregulate plasminogen activator inhibitor-1 (PAI-1) in platelets and endothelial cells, leading to impaired fibrinolytic function (Lipinski et al., 2011). This bidirectional interaction between the inflammatory and coagulation systems is a key factor that determines the high thrombogenicity of atherosclerotic plaques.

It is worth noting that LPS released by the microbial communities that are enriched in the high-altitude environment may promote the occurrence of cardiovascular complications in patients with certain diseases. A large-scale multi-omics study has shown that *Prevotella copri* is one of the dominant bacterial species in the gut microbiota of populations in high-altitude hypoxic regions, suggesting that it may be involved in the host’s adaptation to high-altitude environments (Qi et al., 2025). However, studies have shown that in the chronic kidney disease model, *Prevotella copri* can activate the TLR4/NF-κB/NLRP3 inflammatory signaling pathway through its derived LPS, thus also promoting the osteogenic differentiation of vascular smooth muscle cells and vascular calcification (Hao et al., 2024). This indicates that for those who are simultaneously in a high-altitude hypoxic environment and have chronic kidney disease, their gut microbiota, especially the genus *Prevotella copri*, may be a potential element that could have an impact on the risk of cardiovascular complications. The dual characteristics of *P. copri* in the high-altitude environment reflect the distinct species and functional differences within the genus Prevotella. Specifically, certain strains of the genus Prevotella (such as *P. histicola*) exert anti-inflammatory and barrier-protective effects by inducing regulatory T cells (Marietta et al., 2016). Conversely, species such as the aforementioned *P. copri* and the oral-derived *P. intermedia* can drive inflammation and disrupt the intestinal barrier via the TLR/NF-κB pathway (Hao et al., 2024; Glavina et al., 2026). This difference within the genus indicates that in healthy individuals living at high altitudes, low-acetylated LPS or short-chain fatty acids generated by the normal Prevotella may play a part in metabolic adaptation and immune balance (Zhao et al., 2024; De Simone Carone et al., 2025). However, when the host is already suffering from a disease (such as chronic kidney disease or rheumatoid arthritis), Prevotella species with pro-inflammatory phenotypes are selectively promoted. The LPS released from high-altitude stimulation has a higher probability of getting into the circulatory system via the damaged intestinal barrier, thereby intensifying the original disease and possibly initiating cardiovascular diseases (Scher et al., 2013; Hao et al., 2024). Therefore, for individuals simultaneously exposed to high-altitude hypoxic environments and suffering from underlying diseases, the final function of the Prevotella microbiota in their intestines is not simply determined by the quantity at the genus level, but is determined by a dynamic functional combination that is determined by the composition of species within the species, the host immune state, and the intestinal microenvironment together.

Meanwhile, the LPS inflammatory pathway mediated by the imbalance of gut microbiota may also cause more obvious cardiovascular pathological effects in newborns and infants living in high-altitude areas, especially those little ones with congenital heart disease (CHD). A high-altitude hypoxic environment is regarded as a potential environmental risk factor for CHD. A long-term observational study conducted in Ecuador shows a strong positive correlation between the prevalence of CHD and altitude; the national average prevalence was 70.6 cases per 10,000 live births, while in areas above 3,000 meters in altitude, the prevalence could rise to over 89 cases per 10,000 (González-Andrade, 2020). Similarly, in a census carried out on over 80,000 school-aged children in the Nagqu area of Tibet in China, where the average altitude is over 4,000 meters, found that the overall CHD prevalence was 5.21‰, with prevalence increasing in a stepwise manner with altitude, reaching 8.12‰ in areas above 4,500 meters (Chun et al., 2019). Preliminary clinical observations have revealed that newborns with CHD complicated by hypoxia (oxygen saturation <90%) tend to exhibit early-stage gut microbiota dysbiosis, which is characterized by an increase in the relative abundance of potential pathogenic bacteria (like Streptococcus and Pseudomonas) and a decrease in beneficial bacteria (like Bifidobacterium) (Renk et al., 2025). In early life, the host’s intestinal barrier and immune system, which are still in the process of development, are extremely vulnerable to the hypoxia and microbial dysbiosis that occur in the pathophysiological process of CHD. This state, by continuously activating the immune-inflammatory pathway, may influence the progression, the treatment response, and the long-term prognosis of CHD (Liu X.-F. et al., 2023). However, this hypothesis still requires validation through longitudinal cohort studies.

## 4 Cardiovascular adaptive protective mechanisms of the gut microbiota in high-altitude environments

### 4.1 SCFA metabolic adaptation: activation of core protective pathways

**Figure 2:**
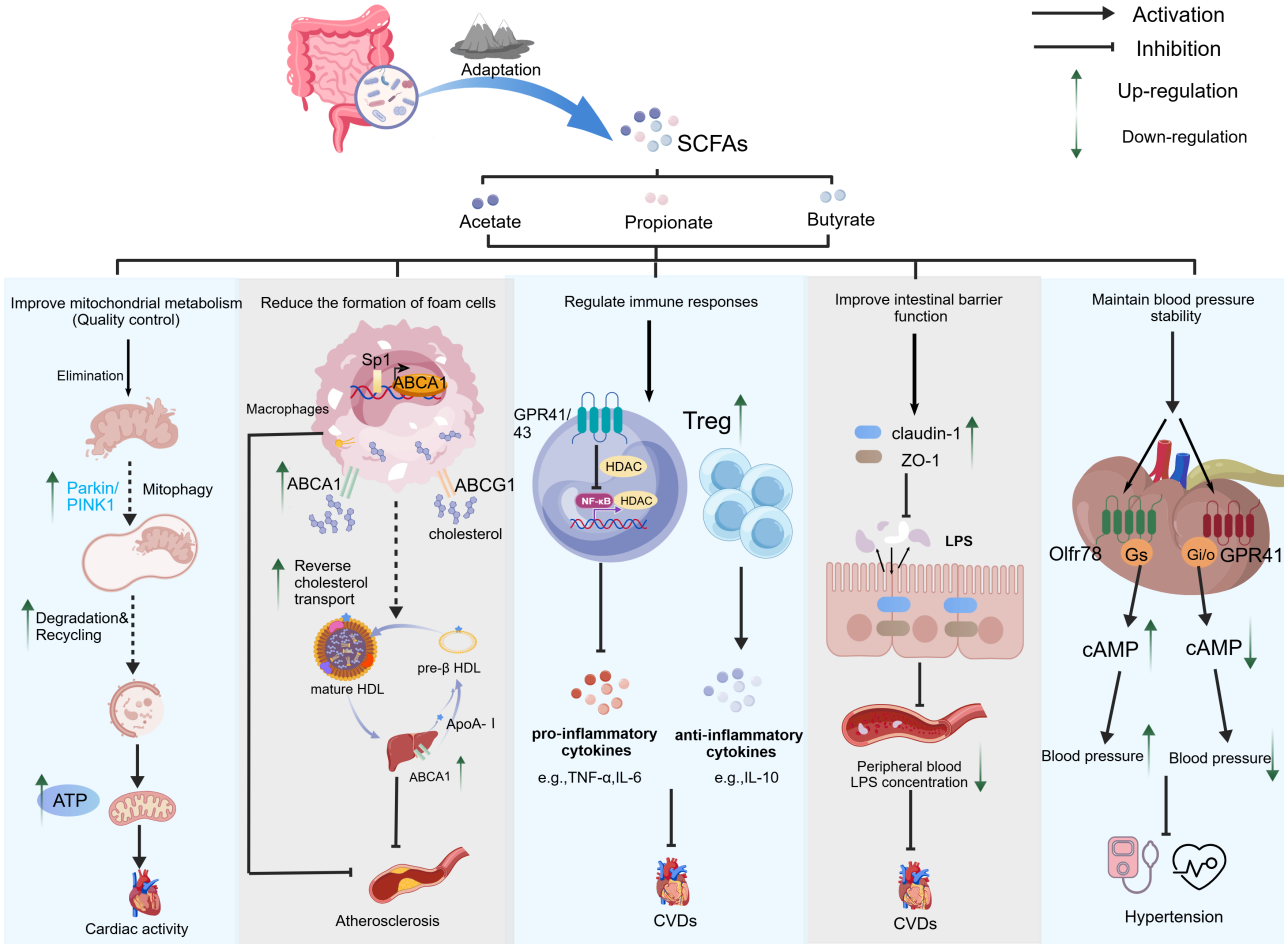
Cardioprotective effects of gut microbiota-derived short-chain fatty acids (SCFAs) during high-altitude adaptation. SCFAs exert multi-faceted beneficial effects to inhibit cardiovascular disease progression, the main mechanisms are as follows: (1) promote aging and mitochondrial degradation/recovery under stress through the Parkin/PINK1 pathway, thereby optimizing mitochondrial metabolism and myocardial energy metabolism; (2) promote the reverse transport of cholesterol to reduce the formation of foam cells; (3) inhibit inflammation through the GPR41/43-HDAC signaling pathway and regulatory T cell polarization; (4) enhance intestinal barrier integrity by upregulating tight junction proteins (claudin-1, ZO-1) and mucin secretion; (5) renin-mediated regulation of blood pressure fluctuations through olfactory receptor-mediated mechanisms. Created with BioGDP.com.

The SCFAs generated through the fermentation of dietary fiber by the gut microbiota, which mainly consist of acetate, propionate, and butyrate, are the key metabolites taking part in cardiovascular protection in high-altitude regions. However, the quantity of their producing bacteria decreases under acute high-altitude exposure (Table 1), and research shows that during the second week of high-altitude exposure in healthy people, the concentrations of total SCFAs, acetate, propionate, and butyrate in feces significantly decrease (Karl et al., 2018). However, SCFAs are proposed to exert a protective function when human beings or other mammals encounter high-altitude challenges, which is a crucial manifestation of the adaptive reaction of the gut microbiota to high-altitude exposure: Zhao et al. (2024) identified through a meta-analysis that the guts of high-altitude-adapted individuals are rich in butyrate-producing bacteria and exhibit higher abundances of key genes involved in butyrate synthesis within their gut microbiota. The genus Prevotella of the Tibetan population is significantly enriched (Table 1), which is a genus mainly producing acetate and propionate (Han et al., 2024). Animals living at high altitudes also exhibit enrichment of SCFAs and the producing bacterial communities (Liu N. et al., 2021). Among humans and animals that adapt to high altitudes, there is a wide enrichment in SCFAs and their producing bacteria, which indicates that SCFAs are representative metabolites through which the gut microbiota mediates the body’s adaptation to high altitudes (Figure 2).

In the high-altitude environment, the relatively scarce food resources combined with hypoxia will reduce the energy metabolism efficiency. However, after long-term adaptation, high-altitude organisms show significant enrichment of bacterial genera capable of producing SCFAs (Table 1), which indicates that the gut microbiota improves the energy intake ability by adjusting the community composition and then provides metabolic support for the cardiovascular system. SCFAs can improve the myocardial dysfunction that originates from high-altitude hypoxia by adjusting myocardial energy metabolism pathways. In a heart transplant-related ischemia/reperfusion (I/R) injury model, butyrate can restore oxidative phosphorylation and adenosine triphosphate (ATP) production by inhibiting histone deacetylation, promoting Parkin/PINK1-mediated mitochondrial autophagy, and assisting in mitochondrial biosynthesis to replace the damaged mitochondria (Ruidas et al., 2026). Carley et al. (2021) confirmed that in the failing heart, the degree of oxidation of butyric acid is greater than that of ketone bodies, exhibiting a higher oxidation rate. Panagia et al. (2020) also confirm that butyrate can effectively rectify the defects of the myocardial energy metabolism, doubling the reduced mitochondrial ATP synthesis rate in damaged hearts. Quantitative metabolomics research shows that the human heart actively takes up acetic acid (accounting for 2% of myocardial carbon uptake), and the consumption of acetate is in proportion to its circulating concentration (Shi and Qiu, 2022), which suggests that acetate directly provides energy to the myocardium. Propionate is transformed into glucose within the liver, which provides energy to the heart and skeletal muscles. SCFAs that can reduce the levels of lipid peroxidation in post-injury cardiomyocytes resulting from energy metabolism problems caused by hypoxia can enhance the survival rates of cardiomyocytes (He et al., 2024). Under certain conditions, propionate, which makes the myocardial energy efficiency better by triggering cardiac metabolic reprogramming, inhibiting fatty acid oxidation, and promoting glucose utilization (Wang et al., 2018). Furthermore, butyrate makes macrophages excrete cholesterol by upregulating the expression of the ATP-binding cassette transporter A1 (ABCA1), thus reducing the lipid accumulation in atherosclerotic plaques as well (Du et al., 2020).

SCFAs can directly strengthen the intestinal epithelial barrier function and exert immune regulatory effects. In an AML mouse model, the intervention of butyrate can repair the intestinal barrier damage by up-regulating the expression of tight junction proteins claudin-1, ZO-1, and occludin, significantly reducing intestinal permeability and effectively lowering plasma LPS level (Wang Y. et al., 2022). Meanwhile, butyrate significantly reduces the activity of lactate dehydrogenase A (LDHA) and lactate levels in hypoxic cells, downregulates the overexpression of hypoxia-induced HIF-1*α*, and prevents the intestinal epithelial barrier damage caused by the excessive activation of the HIF-1α pathway (Zhao et al., 2024). In response to pathogenic bacteria, SCFAs can promote the secretion of antimicrobial peptides such as RegIIIγ and *β*-defensin by intestinal epithelial cells through the G protein-coupled receptor GPR43 (Zhao et al., 2018), while inhibiting the excessive growth of various intestinal pathogens, including *Escherichia coli* and *Klebsiella pneumoniae*. This is helpful for restoring the disrupted microbial balance and also for reducing endotoxin production (Chang et al., 2024). As important immunomodulatory signals, SCFAs inhibit the production of pro-inflammatory cytokines by activating GPR41/43 receptors and inhibiting histone deacetylases, thus preventing unfavorable myocardial remodeling and contractile dysfunction (Liu Y. et al., 2023; Orjichukwu et al., 2025). At the same time, they directly promote the proliferation and anti-inflammatory functions of regulatory T cells (Tregs) to increase the production of IL-10 (Smith et al., 2013) and act on γδ T cells to inhibit the production of IL-17 (Dupraz et al., 2021), which is crucial for maintaining the balance of mucosal immunity. Research by Bartolomaeus et al. (2019) demonstrated that propionate could reduce cardiac immune cell infiltration and fibroblast activation by promoting Treg function and suppressing pro-inflammatory T-cell responses, thereby improving cardiac remodeling and significantly alleviating cardiac hypertrophy and myocardial fibrosis. Research by Hu et al. (2014) indicates that butyric acid, through inhibiting the expression of HMGB1 protein, notably reduces the levels of TNF-α and IL-6 in the myocardium, while reducing the area of myocardial infarction and suppressing the accumulation of oxidative stress marker malondialdehyde (MDA), and boosting the activity of superoxide dismutase (SOD). Butyrate can also reduce the expression of inflammatory factors in human endothelial cells by inhibiting the NF-κB signaling pathway, and decreases the adhesion of THP-1 monocytes to endothelial cells (Aguilar et al., 2014). In an ApoE−/− mouse model, a dietary supplement with 1% sodium butyrate reduces the area of atherosclerotic lesions in the aorta by 50% and enhances plaque stability more enhanced (Aguilar et al., 2014).

In the mouse model, SCFAs have been confirmed to exert a bidirectional regulatory effect on blood pressure by acting on the paired G protein-coupled receptors Olfr78 and GPR41. Specifically, SCFAs promote renin release by activating the Olfr78 receptor expressed in the kidneys, producing a blood pressure-raising effect; conversely, they mediate vasodilation by activating the GPR41 receptor, producing a blood pressure-lowering effect (Pluznick et al., 2013). The synergy of these two opposite actions provides a molecular basis for buffering the blood pressure fluctuations caused by the physiological fluctuations in SCFA concentrations. Deng et al. (2022) also utilized mouse model to demonstrate that propionate, by activating GPR41, reduces the level of Ang II and regulates the expression of the caveolin-1/angiotensin-converting enzyme 2 axis, thereby alleviating the myocardial ischemia/reperfusion injury that is aggravated by Ang II. In the mouse model of hypertension induced by Ang II infusion, oral propionate obviously reduced the cardiac hypertrophy indices and myocardial fibrosis (Bartolomaeus et al., 2019). Robles-Vera et al. (2020) demonstrated in in experiments on rat aortic endothelial cells (RAECs) that butyrate and acetate effectively antagonize the Ang II-induced endothelial oxidative stress and reduce nitric oxide bioavailability, thereby improving vascular endothelial function. Moreover, *in vitro* and rodent studies suggest that propionates may relieve the dysfunction of coronary microvascular endothelial cells caused by ox-LDL, which is realized by inhibiting endoplasmic reticulum stress, preventing the phosphorylation of eNOS, enhancing the bioavailability of NO, while inhibiting the proliferation of vascular smooth muscle cells to maintain the vasodilatory function (Tsihlis et al., 2011; Hong et al., 2024). However, the clinical trial results about the role of SCFAs supplements in human hypertension are rather controversial and have not reached a consensus so far. A prebiotic intervention aimed at increasing colonic SCFAs has shown a moderate effect of lowering blood pressure in hypertensive patients (Jama et al., 2023); however, another double-blind randomized controlled trial reported that direct oral supplementation of sodium butyrate did not lower systolic and diastolic blood pressure in humans, but instead led to an increase in blood pressure (Verhaar et al., 2024). The differences between these two researches might be related to the administration ways, the absorption positions and the action mechanisms. Therefore, there is an urgent need for large-scale, long-term clinical trials combined with multi-omics analysis to figure out the exact role of the SCFA receptor signaling pathway in human blood pressure regulation, especially when being exposed to high altitudes.

Preclinical studies in rodent models have demonstrated that butyrate can exert cardioprotective effects through the gut-brain neural circuit, especially through the ways that depend on the vagus nerve. This mechanism may involve the neural activity of the vagus nerve inhibiting the paraventricular nucleus of the hypothalamus (PVN) and the superior cervical ganglion (SCG), thereby regulating the cardiac sympathetic tone (Yu et al., 2021). In myocardial I/R injury model of these animals, the supplement of butyrate can significantly reduce the area of myocardial infarction and also decrease the levels of myocardial injury markers in plasma, including lactate dehydrogenase (LDH), creatine kinase (CK), and its isoenzyme CK-MB (Yu et al., 2021). Although at present the exact neural pathways for human heart protection in high-altitude areas have not been tested out, preliminary clinical evidence is constantly accumulating showing that SCFAs probably regulate the human central nervous system and stress response through the gut-brain axis. For example, a randomized, placebo-controlled trial has confirmed that colon-delivered SCFAs have significantly reduced the cortisol response of healthy males to psychosocial stress, which indicates that microbial metabolites may regulate the central stress circuit (such as the hypothalamic–pituitary–adrenal (HPA) axis) (Dalile et al., 2020). However, it is worth noting that hypoxia itself is a potent activator of the sympathetic nervous system (Duplain et al., 1999). Therefore, the interactions between high-altitude hypoxic stress, SCFAs (such as butyrate), and the autonomic nervous system require further validation.

### 4.2 Adaptive uric acid metabolism

Chronic hypoxia can lead to tissue ischemia or hypoxia as well as oxidative stress, accelerating purine degradation and increasing uric acid production (Zhou et al., 2024). Exposure to the hypoxic environment at high altitude will greatly change the intestinal purine metabolism pathway, and the gut microbiota promotes host adaptation to high altitude by reprogramming uric acid metabolism. Su et al. (2024b) conducted a long-term longitudinal study on healthy males who relocated from a low altitude (243 m) to a high altitude (3,658 m). It was discovered that during the adaptation process, the abundance of gut bacteria that carry the uric acid degradation gene cluster is significantly increased. These bacteria primarily belong to the family Lachnospiraceae (e.g., Blautia A and Enterocloster), and Collinsella can also be regarded as one of the main carrier genera. It is worth noting that although the uric acid levels in the intestine initially rise after exposure and then decline, reaching undetectable levels in long-term high-altitude residents; meanwhile, the abundance of uric acid-degrading bacteria increases during exposure and remains at a relatively high level over the long term. These concurrent trends indicate that the reduction of intestinal uric acid levels may be functionally related to the enrichment of these degrading bacteria. A multi-omics study by Han et al. (2024) confirmed that in the gut microbiota of the Tibetan population native to the plateau, the genes of key enzymes involved in purine synthesis, purF and purH, are upregulated, while purine degradation genes are downregulated. Meanwhile, the abundance of bacterial strains such as *Escherichia coli* and *Klebsiella pneumoniae* that participate in purine degradation by secreting xanthine oxidase (XOD) was reduced in the gut of high-altitude populations, forming a dual regulatory mechanism to regulate the host plasma uric acid levels, resulting in a significant reduction in uric acid levels in the host’s blood. The deposition of uric acid crystals is an important inducer of vascular injury at high altitudes. Uric acid can be oxidized by peroxidases such as peroxidasin (PXDN), generating reactive intermediate products, which causes the imbalance of redox in endothelial cells. Additionally, it can activate endothelial cells, upregulate the expression of adhesion molecules such as intercellular adhesion molecule 1 (ICAM-1) and vascular cell adhesion molecule (VCAM-1), promote monocyte adhesion, and also induce a pro-inflammatory phenotype (Dempsey et al., 2025). Uric acid can also cause vascular endothelial dysfunction through mechanisms like activating the NLRP3 inflammasome and inducing endothelial-mesenchymal transition (Yang and Kong, 2020). Studies suggest that uric acid may also enter cells through transporters such as urate transporter 1 (URAT1)/glucose transporter 9 (GLUT9), and would also have an impact on the bioavailability of nitric oxide (NO) (Dempsey et al., 2025). The alterations in the gut microbiota at high altitudes and the subsequent reprogramming of uric acid metabolism, which implies that the microbiota might relieve vascular injury by regulating host uric acid homeostasis, have\ turned into one of the crucial mechanisms promoting high-altitude adaptation.

### 4.3 Adaptive regulation of inflammation and immunity

High-altitude hypoxia can disrupt intestinal barrier integrity and trigger systemic inflammation, while the gut microbiota can alleviate cardiovascular inflammatory damage by enriching anti-inflammatory strains, strengthening the intestinal barrier, and regulating immune balance. Bacterial strains having anti-inflammatory effects, like Bifidobacterium, Lactobacillus, and Parabacteroides, are significantly enriched in the intestines of humans and animals adapted to high altitudes (Table 1). *Bacillus subtilis* strains BS1 and BS2 isolated from the intestines of yaks on the Tibetan Plateau at an altitude of 3,600 m can raise the level of anti-inflammatory factor IL-10 in the serum of mice, significantly downregulate pro-inflammatory factors TNF-α, IL-6, and IL-8, and at the same time can enhance the expression of serum antibodies IgG, IgM, and IgA (Li Z. et al., 2019). A recent study have identified a protein named DUF4925 from *Parabacteroides distasonis*, which can directly bind to LPS, effectively blocking the interaction between LPS and the TLR4 receptor, and inhibiting LPS-induced pro-inflammatory cytokine production and NF-κB signaling pathway activation in a dose-dependent way (Guo et al., 2026). Experiments conducted by Wan et al. (2022) on mice in high altitude show that the intervention with probiotic *Lactobacillus johnsonii* YH1136 which is isolated from the feces of healthy Tibetans at an altitude of 5,000 meters can reduce the levels of intestinal inflammatory factors, can also improve the intestinal barrier function, while inhibiting the proliferation of the opportunistic pathogen Staphylococcus, restoring the dominant position of Lactobacillus, and then preventing the intestinal dysfunction which is caused by endogenous pathogens. *Bifidobacterium longum* JBLC-141 can alleviate oxidative stress and inflammatory responses by activating the KEAP1/NRF2 pathway, thereby improving hypoxia-induced destruction of intestinal tight junctions, apoptosis, and dysbiosis, and thus it has a comprehensive protective effect on the intestinal barrier in high-altitude hypoxic environments (Li X.-Y. et al., 2024). Particular *Bifidobacterium bifidum* strains, like LMG13195, can promote the maturation of dendritic cells, and thus cause the differentiation of naive CD4 + T cells into Tregs (López et al., 2011). These strains can also activate memory immune responses and stimulate IL-17 production, supporting the view that the Treg/Th17 cell population is plastic, suggesting that commensal bacteria may maintain mucosal tolerance by inducing Tregs (López et al., 2011). *Akkermansia muciniphila*, which is a key biomarker of gut health, is found to be in a higher abundance in the guts of populations adapted to high altitudes (Table 1). The extracellular vesicles from *Akkermansia muciniphila* can mediate the increase in occludin expression, which is dependent on AMP-activated protein kinase (AMPK), thereby also strengthening intestinal epithelial tight junctions (Chelakkot et al., 2018); its outer membrane protein Amuc_0904 directly binds to and inhibits the phosphorylation of the MET receptor, downregulating the Wnt/*β*-catenin signaling pathway, while enhancing oxidative phosphorylation in epithelial cells, thereby promoting goblet cell differentiation and the production of the mucin protein MUC2, and also strengthening the integrity of the intestinal barrier to maintain intestinal homeostasis (Yang M. et al., 2025). In ApoE⁻/⁻ mice, *Akkermansia muciniphila* intervention can inhibit the formation of atherosclerotic lesions induced by LPS entry into the bloodstream by enhancing intestinal barrier integrity (Li K. et al., 2016). A study by Zhu et al. (2020) specifically compared the gut microbiota of hypertensive patients at different altitudes, finding that the abundance of the genus Akkermansia was significantly higher in high-altitude Tibetan and mid-altitude Han Chinese hypertensive patients than in low-altitude populations and was negatively correlated with cardiovascular disease and inflammatory responses.

### 4.4 Adaptive strategies for alleviating obesity and type 2 diabetes

Obesity and type 2 diabetes are the main risk factors of cardiovascular diseases. Among the general population, obese individuals have an approximately 60% higher likelihood of contracting coronary heart disease compared with those of normal weight. Moreover, for every 1-unit increase in body mass index (BMI), the risk ratio for coronary heart disease rises to 1.04 for women and 1.05 for men (Bhupathiraju and Hu, 2016). Cardiovascular disease is the primary cause of death among diabetic patients, accounting for 44 and 52% of deaths in type 1 and type 2 diabetic patients, respectively (Morrish et al., 2001). Additionally, adult diabetic patients have a risk of death from myocardial infarction, ischemic heart disease, congestive heart failure, and stroke that is 2 to 4 times higher compared to non-diabetic populations (Matheus et al., 2013). The gut microbiota plays a key role in the pathogenesis of obesity, type 2 diabetes, and cardiovascular disease, influencing host metabolic health through pathways such as SCFA metabolism, bile acid transformation, and inflammation regulation (Marzullo et al., 2020; Li et al., 2017; Alhajri et al., 2021). A study regarding the gut microbiota of Tibetan children who reside in high-altitude regions indicates that the children within the high- altitude obesity group have notably lower levels of serum triglyceride (TG) and low-density lipoprotein cholesterol (LDL-C) levels compared to those in the low-altitude obese group. Analysis of the gut microbiota indicates that the relative abundance of Prevotella is positively correlated with altitude and is significantly enriched in the intestines of children in the high-altitude group; such enrichment of Prevotella may be related to the lower prevalence of obesity observed in the high-altitude environment (Du et al., 2022). In a study of Tibetan populations living at different altitudes, it was discovered that BMI, waist circumference, and waist-to- height ratio all decrease as altitude increases, and the overall prevalence of obesity shows a similar tendency (Sherpa et al., 2010). Studies have shown that intermittent hypoxia can modify the composition of the gut microbiota of overweight or obese males, and it significantly increases the relative abundance of butyrate-producing obligate anaerobic bacteria, such as Fusicatenibacter and Butyricicoccus. What is clearly related are these changes in the microbial composition and the changes in various metabolic indicators, including the insulin sensitivity of the host adipose tissue, the peripheral insulin sensitivity, and the plasma fatty acids (Van Meijel et al., 2022). Butyrate promotes energy expenditure, mitochondrial biogenesis, and fatty acid oxidation by activating the AMPK and p38 MAPK pathways, and then upregulates the activity and expression of peroxisome proliferator-activated receptor Gamma Coactivator-1α (PGC-1α), which is beneficial for preventing and treating diet-induced obesity and improving insulin sensitivity (Gao et al., 2009). Additionally, as an HDAC inhibitor, butyrate upregulates the expression of multiple diabetes-related genes, including the insulin receptor (INSR), which implies that butyrate may enhance the transcriptional activity of the insulin pathway through HDAC inhibition (Shapira et al., 2025). After the adaptation to high altitude, there is an increase in the quantity of *Akkermansia muciniphila*, which can also improve insulin sensitivity and reduce the obesity rate. In a high-fat diet-induced obese mouse model, daily oral administration of live *Akkermansia muciniphila* for 4 consecutive weeks obviously reversed the diet-induced weight gain and fat accumulation and also improved various metabolic disorders, including fasting hyperglycemia, insulin resistance, and metabolic endotoxemia (Everard et al., 2013). Studies have also confirmed that *Akkermansia muciniphila* and its secreted protein P9 can induce glucagon-like peptide-1 secretion and cause brown adipose tissue to generate heat, thereby improving glucose homeostasis and alleviating metabolic diseases in mice on a high-fat diet (Hs et al., 2021).

## 5 Strategies for the prevention and treatment of cardiovascular diseases at high altitude based on gut microbiota regulation

### 5.1 Dietary intervention

Diet is one of the most direct and effective environmental factors shaping the structure and function of the gut microbiota. The implementation of structured dietary intervention strategies that are tailored to the characteristics of high-altitude environments is aimed at optimizing the microbial composition, promoting the production of protective metabolites, suppressing harmful pathways, and thus establishing the first line of defense against cardiovascular damage. Because the gut microbiota of low-altitude migrants only approximates that of native high-altitude populations, dietary intervention represents the most feasible approach to narrowing this gap. The exploration and promotion of diets with high-altitude characteristics possess significant value. Populations living long-term in high-altitude regions have incorporated rich adaptive wisdom into their traditional diets. For example, traditional Tibetan fermented dairy products like yogurt and milk, which are full of active lactic acid bacteria when consumed, can help to enrich beneficial bacterial genera such as Bacteroides and Faecalibacterium in the intestines and then promote the production of SCFAs (Liu et al., 2020). Similarly, using grains rich in *β*-glucan, like highland barley, as raw materials can increase the intake of fermentable dietary fiber and thus provide a fermentation substrate for gut microbes. *In vitro* fermentation studies show that this can effectively regulate the microbial composition and significantly increase the total production of SCFAs, including butyrate, which has powerful anti-inflammatory and cardioprotective effects (Ge et al., 2025). In addition, certain functional plant components that are peculiar to high-altitude regions also show the potential of regulating the microbiota. For example, the polysaccharide that exists in large quantities in the *Brassica rapa* L. has been shown *in vitro* and in animal models to regulate the gut microbiota. It can enrich beneficial bacteria like *Akkermansia muciniphila* and *Leuconostoc lactis*, raise the level of short-chain fatty acids, especially butyric acid, and also repair the gut barrier function by enhancing the expression of tight junction proteins and increasing the number of goblet cells, thereby relieving oxidative stress and intestinal inflammation (Liu et al., 2024).

The adjustment to the basic dietary pattern forms the cornerstone of intervention, and the core principle is a diet with high dietary fiber, low saturated fat, and added sugar. A diet with a high fiber content, especially resistant starch such as high-amylose corn starch, offers sufficient fermentable substrates for the colonic commensal bacteria, specifically stimulating the growth of butyrate-producing bacteria like *Faecalibacterium prausnitzii* and Roseburia (Maier et al., 2017). At the same time, reducing the intake of red meat, processed meats, and high-fat dairy products can decrease the dietary supply of choline and L-carnitine, thereby reducing from the source the metabolism of these substances by intestinal microorganisms such as Clostridium spp. that are rich in choline TMA-cleaving enzymes CutC/D into TMA, and then reducing the level of its hepatic oxidation product TMAO in order to alleviate the risk of atherosclerosis and thrombosis (Zhang et al., 2025). A low-sugar diet can suppress the excessive growth of pro-inflammatory Proteobacteria like Enterobacteriaceae, while supporting the growth of Bacteroidetes that have anti-inflammatory and barrier-protective functions, thus maintaining the balanced state of intestinal immunity (Satokari, 2020). Furthermore, the promotion of food consumption that is full of polyphenols and antioxidants, such as berries, nuts, dark-colored vegetables, and black chocolate, and so on, which are helpful for alleviating the oxidative stress situation that is aggravated by the combined action of high-altitude hypoxia and dysbiosis (Koivisto et al., 2019). For example, when athletes are carrying out high-altitude training, such dietary interventions can increase the level of antioxidants in plasma and can also reduce to a certain degree the inflammatory response brought about by hypoxia (Koivisto et al., 2019). In addition, a large number of studies carried out with cell models indicate that polyphenolic compounds like flavonoids, which possess antioxidant, anti-apoptotic, and anti-inflammatory properties, can effectively relieve myocardial ischemia–reperfusion injury, thus offering protection to the cardiovascular system (Ali et al., 2021).

### 5.2 Probiotic/prebiotic/synbiotic interventions

On the foundation of diet adjustment, directly supplementing exogenous beneficial microorganisms, probiotics, or their selective substrates, prebiotics, or even synbiotics, which combine both, this is a core biological intervention strategy for the precise operation on the gut microbiome in order to prevent and treat cardiovascular diseases at high altitudes.

The selection of probiotic strains and clinical evidence is key to the success of such interventions. Priority should be given to those strains that have already demonstrated cardiovascular protection potential in clinical research or those strains that have performed well in high-altitude adaptation models. For example, supplementation with specific Lactobacillus like *Lactobacillus plantarum*, and Bifidobacterium, such as *Bifidobacterium bifidum*, strains has shown a lowering effect on LDL-C in randomized controlled trials or animal models (Fuentes et al., 2013; Bordoni et al., 2013). More importantly, research evidence shows that supplementing probiotics and synbiotics for adults with prediabetes and type 2 diabetes can notably lower their systolic and diastolic blood pressure (Setayesh et al., 2026), which is directly related to dealing with the common hypertension risks in high-altitude environments. Studies have also indicated that taking a large amount of *Akkermansia muciniphila* orally for a long period is feasible; specifically, the heat-inactivated form of *Akkermansia muciniphila* can safely improve insulin sensitivity, reduce cholesterol, and also reduce systemic inflammation, providing a positive metabolic regulation potential for overweight/obese patients with insulin resistance (Depommier et al., 2019). In recent years, “second-generation probiotics” isolated from the intestines of healthy individuals have demonstrated significant potential. For example, *Parabacteroides merdae* has been shown to improve glucose homeostasis and inhibit atherosclerosis by enhancing the catabolism of branched-chain amino acids (Wang H. et al., 2025). Meanwhile, in animal models, it can be observed that the co-supplementation of *Bacteroides cellulosilyticus*, *Faecalibacterium prausnitzii*, and *Roseburia intestinalis* will reshape the microbiota and promote the conversion of secondary bile acids, thereby leading to the reduction of blood lipids and the inhibition of the progression of atherosclerosis (Jie et al., 2023).

Prebiotics are food components that are not digested or absorbed by the host but selectively stimulate the growth and activity of one or a few beneficial bacteria in the gut (Yeo et al., 2009). Indigestible dietary fibers, such as inulin and fructooligosaccharides, can act as selective prebiotics, which are fermented and utilized by beneficial gut bacteria such as Bifidobacterium. This fermentation not only directly promotes the growth of Bifidobacterium and other bacterial populations, but its metabolic products can also, through a “cross-feeding” mechanism, further stimulate the proliferation and metabolic activity of acetate-consuming and butyrate-producing bacteria like *Faecalibacterium prausnitzii*, thereby significantly increasing the production of SCFAs in the gut (Yeo et al., 2009; Kim et al., 2020).

Synbiotic interventions combine the benefits of probiotics and prebiotics. Commercially available synbiotic formulations typically contain a combination of multiple live bacterial strains and prebiotics. Clinical studies have shown that in overweight patients with coronary heart disease and type 2 diabetes, supplementing with a synbiotic preparation containing *Lactobacillus acidophilus*, *Lactobacillus casei*, and *Bifidobacterium bifidum*, along with inulin, can significantly reduce fasting blood glucose, improve insulin metabolism-related markers such as serum insulin levels, and increase high-density lipoprotein cholesterol (HDL-C) levels (Tajabadi-Ebrahimi et al., 2016). Further systematic review evidence indicates that the supplementation of probiotics or prebiotics has anti-inflammatory and antioxidant effects among coronary heart disease patients, significantly reducing inflammatory markers such as serum high-sensitivity C-reactive protein (hs-CRP) and oxidative stress markers like MDA (Yang et al., 2026). Furthermore, the combination of probiotics and conventional cardiovascular drugs, such as statins, has shown potential for synergistic effects. It can well optimize the composition of the intestinal flora of patients with hyperlipidemia by increasing beneficial bacteria and reducing harmful bacteria (Tian et al., 2024). The metabolic regulatory potential of the Probiotic/Prebiotic/Synbiotic described above is mainly based on evidence originating from low-altitude populations or animal models; their strain adaptability in high-altitude environments, optimal dosages, and long-term safety still need to be verified through randomized controlled trials carried out in high-altitude populations.

### 5.3 Fecal microbiota transplantation and targeted strain intervention

When dietary adjustments and microbiome modulators such as probiotics and prebiotics yield limited results, FMT and targeted functional strain transplantation involve introducing specific functional strains—derived from strictly screened healthy donors or specific strain combinations—into patients (Fan et al., 2025). These strains possess the potential for metabolite-targeted and cardiovascular regulation, aiming to achieve more durable and effective treatment. Ideal donors should be healthy individuals with good adaptability to high-altitude environments. Recent studies have revealed associations between the unique structure of the gut microbiota in populations adapted to high-altitude environments and their metabolic phenotypes. For example, Li Z. et al. (2025) found that the gut microbiota of Han Chinese populations, who had migrated to high-altitude regions, was enriched with Prevotella, and that the functional profile of their microbiota was associated with changes in lipid metabolism pathways. Furthermore, compared to low-altitude populations, the gut microbiota of Tibetan individuals living at high altitudes and Han Chinese hypertensive patients living at moderate altitudes exhibit higher microbial diversity, a lower F/B ratio, and an enrichment of the genus Akkermansia. These characteristics are believed to contribute to physiological adaptation to the high-altitude environment by regulating host inflammatory responses and energy metabolism and may exert positive effects on cardiovascular metabolic parameters (Zhu et al., 2020). Therefore, screening such populations as donors, or further identifying donors whose gut microbiota is clearly associated with cardiovascular protective phenotypes (such as an optimal lipid profile, low inflammation levels, and good vascular function) through multi-omics analysis, is key to improving the efficacy of FMT. Animal studies have confirmed that transplanting fecal microbiota from plateau zokors—animals adapted to high-altitude environments—into recipient animals exposed to low altitudes can significantly improve the latter’s hypoxic tolerance and alleviate elevated pulmonary artery pressure and right ventricular hypertrophy (Chen Z. et al., 2025). This mechanism is related to an increase in the levels of beneficial metabolites such as butyrate and ILA in the receiver’s gut, as well as a decrease in inflammatory responses. More precisely, the transplantation aimed at bacterial strains is also promising. For instance, the transplantation of the single-strain *Blautia wexlerae* has been proven to relieve the damage to the cardiopulmonary tissues in the mouse model exposed to high altitude; this effect is closely related to the reduction in the levels of pro-inflammatory cytokines IL-1*α*/*β* and the increase in the expression of tight junction proteins in the intestinal epithelium (Su et al., 2024a). These studies provide important pre-clinical evidence for the application of FMT and strain-specific therapies in cardiovascular diseases at high altitude.

Although the prospects are promising, the application of FMT in the prevention and treatment of cardiovascular diseases at high altitudes still faces challenges, including the need for standardization of donor screening criteria, the selection of transplantation routes like oral capsules colonoscopy, the need for evaluation of long-term safety and efficacy, and potential ethical issues (Zou et al., 2022; Grigoryan et al., 2020; Kao et al., 2017). Future research may focus on developing synthetic microbiota based on the characteristics of “super-donor” microbiomes, which are composed of multiple identified beneficial strains in specific proportions to replace complex and variable whole feces, thereby improving the controllability, safety, and reproducibility of treatment (Khan et al., 2022). However, at present there are no FMT clinical trials regarding human cardiovascular diseases at high altitude. In the future, it is necessary to carefully screen high-altitude donors having cardiac protective phenotypes and use standardized protocols to assess the safety and efficacy of FMT.

### 5.4 Improvements in lifestyle and environmental adaptation

Apart from the direct microbial intervention, the optimization of the lifestyle as well as the creation of favorable microenvironments, which provide favorable conditions for the colonization and functional expression of beneficial gut microbiota, thereby systematically reducing cardiovascular risk. Gradual ascent and adequate adaptation are the key strategies for preventing acute mountain sickness and the related dysbiosis of gut microbiota. A large-scale longitudinal study shows that acute exposure to rapidly ascending from low altitude (800 m) to extremely high altitude (4,500 m) will trigger severe dysbiosis of gut microbiota, including significant fluctuations of α-diversity, an increase of opportunistic pathogenic bacteria, and a decrease of beneficial bacteria with important physiological functions (Ma X. et al., 2025). What is important is that these changes in the gut microbial community structure caused by acute hypoxic stress still exist even when individuals go back to low-altitude environments, which means that high-altitude exposure may possibly lead to long-term remodeling effects on the gut microbiota. Since it has been shown that acute high-altitude hypoxia can damage the intestinal barrier function, cause dysbiosis, and intensify the inflammatory response, the adoption of a step-by-step, gradual ascending strategy with sufficient rest time at key altitude milestones can indeed provide the necessary adaptation window for the intestinal microbial ecosystem (Qi et al., 2025). This strategy, which can help the microbiota to carry out dynamic adjustments and functional remodeling more smoothly, has the potential to reduce the risk of acute dysregulation and related high-altitude diseases, and assist the body to adapt to the high-altitude environment more smoothly.

Regular and moderate physical exercise can promote beneficial gut microbiota and also enhance cardiovascular health. Research indicates that regular exercise, especially high-intensity exercise, can increase the production of SCFAs in a way related to intensity and greatly improve the metabolic function of the gut microbiota (Reljic et al., 2025). Specifically, high-intensity functional training (HIFT) has been found to significantly enhance the α-diversity of the gut microbiota, enrich beneficial bacteria like Lactobacillus and Lachnospira, while reducing the actinomycetes associated with inflammation (Wang Y. et al., 2025). In the high-altitude environment, the exercise that has been proven to effectively increase the amount of beneficial bacteria such as Roseburia, Bifidobacterium, and *Akkermansia muciniphila*, which are associated with cardiovascular protection as previously mentioned (Min et al., 2024). Exercise-induced gut microbiota optimization can synergistically enhance mitochondrial function and antioxidant capacity (Clark and Mach, 2017; Hoseini et al., 2025). This mechanism might contribute to enhancing the body’s ability to resist oxidative stress and inflammation, thus providing new theoretical perspectives and research directions for exploring its potential role in protecting cardiovascular function under high-altitude hypoxia. The combination of high-altitude cold exposure and exercise will also interact with the gut microbiota. It is discovered that the cold exercise can reverse the gut microbiota changes caused by cold exposure alone, significantly promoting beige fat formation and weight loss, while protecting the cardiovascular system from the adverse effects, such as the rise of cholesterol and triglyceride levels under cold exposure (Meng et al., 2020). But please note that the prescription of exercise intensity at altitude needs to account for the hypoxic ceiling and individual acclimatization status.

Oxygen enrichment and environmental interventions provide external support for maintaining gut microbiota homeostasis at high altitudes. The research indicates that hyperbaric oxygen therapy, through the targeted oxygen enrichment interventions, can effectively regulate gut dysbiosis, increase microbial diversity, and alleviate intestinal and systemic inflammatory responses (Li Y. et al., 2024). For those who have long been living or working in the enclosed high-altitude environment, supplementing oxygen through an oxygen concentrator or a hyperbaric oxygen chamber is a recognized foundational intervention (Song M. et al., 2025). Given that acute high-altitude exposure severely impairs sleep quality, and that sleep disturbances and gut microbiota dysbiosis are closely connected through the bidirectional brain-gut axis, we hypothesize that oxygen-rich intervention to improve sleep may have positive effects on maintaining gut microbiota rhythms, reducing related inflammatory responses, and relieving potential cardiovascular risks (Kan and Zhang, 2025; Song K. et al., 2025). However, at present this mechanism still lacks direct clinical verification, and further research still has to be carried out to confirm the effect of oxygen-rich intervention in improving sleep that can mediate the protection effect on the gut.

### 5.5 Targeted metabolite and drug synergistic interventions

Besides directly regulating the microbial community, it is also possible to adopt precise interventions targeting key metabolites or to carry out synergistic treatments with drugs that are friendly to the intestines. For example, the production of TMAO can be directly inhibited. 3,3-Dimethyl-1-butanol (DMB), which is a structural analog of choline, can non-lethally inhibit those enzymes in gut bacteria that are responsible for producing TMA (such as enzymes like CutC/D), thus effectively reducing the level of TMAO in plasma (Wang et al., 2015). Additionally, DMB can prevent or alleviate the conditions of age-related vascular endothelial dysfunction as well as the increase in aortic stiffness (Brunt et al., 2020; Casso et al., 2022). Recently, research has further uncovered the protective effects of DMB with regard to cardiovascular diseases. It has been confirmed in animal studies that DMB can improve the hemodynamics and pulmonary vascular remodeling of rat models of liliacin-induced pulmonary arterial hypertension (Huang et al., 2022), and it can also delay ventricular remodeling during heart failure by regulating signaling pathways such as TGF-β1/Smad3 (Wang et al., 2020). However, the pharmacokinetic characteristics of DMB in plateau populations and its long-term effects on gut microbiota remain unclear.

When selecting medications for treating cardiovascular diseases or the related symptoms at high altitudes, the potential influence on the gut microbiota ought to be taken into account. Phosphodiesterase-5 inhibitors (PDE5Is; such as sildenafil) can effectively inhibit the rise in pulmonary artery pressure brought about by high-altitude exposure and can also enhance gas exchange; they are regarded as ideal candidate drugs for the prevention and treatment of HAPE (Richalet et al., 2005). Apart from their vascular actions, PDE5Is also exhibit direct cardioprotective properties. Preclinical evidence indicates that these drugs can reduce the infarct size through complex mechanisms and inhibit the adverse myocardial remodeling in heart failure models, like reducing cell apoptosis, interstitial fibrosis, and myocardial hypertrophy (Schwartz et al., 2012). Studies have shown that sildenafil can also reduce mucosal damage and increased permeability in Post-infectious Irritable Bowel Syndrome (PI-IBS) models by activating the cGMP signaling pathway, increasing the density of goblet cells, and upregulating the expression of tight junction proteins (Sharman et al., 2017). This suggests that when drug intervention is necessary for treating cardiovascular diseases at high altitudes, preferentially choosing drugs that have direct protective effects on the intestinal barrier can achieve the dual goals of disease treatment and intestinal protection, and then reduce the risk of drug-induced intestinal injury in the hypoxic environment of high-altitude regions. Conversely, clinicians should exercise caution to avoid unnecessary use of broad-spectrum antibiotics. Although there is research confirming that the short-term use of broad-spectrum antibiotics can significantly reduce plasma TMAO levels by inhibiting the metabolism of precursors like choline by the gut microbiota (Tang et al., 2013); but this effect is temporary, once antibiotics are stopped, the gut microbiota regrows and the level of TMAO will return to normal (Tang et al., 2013; Ding et al., 2025). Furthermore, the long-term or improper excessive use of broad-spectrum antibiotics will indiscriminately kill the gut microbiota, seriously disrupting the intestinal microecological balance. This may lead to long-term dysbiosis, overgrowth of opportunistic pathogens, and exacerbated systemic inflammatory responses, potentially resulting in other adverse effects on cardiovascular outcomes (Ding et al., 2025). Moreover, trials show that antibiotic treatment cannot reduce the cardiovascular mortality of patients with coronary heart disease or lower the risk of adverse events occurring (Andraws et al., 2005). Therefore, based on the current evidence, it is not recommended to use antibiotics as a routine measure for preventing or dealing with cardiovascular diseases that are directed at the gut microbiota. For infectious diseases in high-altitude environments, antibiotics should be used cautiously only when clearly indicated, and subsequent measures to restore the microbiome should be considered.

With the exception of dietary interventions, the scientific basis for the aforementioned gut microbiota-based intervention strategies primarily stems from preclinical studies (cellular/animal models) and trials involving populations at low altitudes. Because of the unique phenomena like gut microbiota remodeling and host metabolic changes that take place in the hypoxic environment of high-altitude areas, the efficacy of these strategies in high-altitude populations, the optimal time of intervention, the dose–response relationship, and long-term safety still need to be verified through large-scale, multi-center randomized controlled trials. At present, these strategies should be considered as promising research directions rather than established clinical practices actually. In the future, it is necessary to integrate the data of the high-altitude group and gradually promote the transformation of the research outcomes from the basic mechanism to the clinical application.

## 6 Conclusions and outlook

### 6.1 Key conclusions

This review systematically explores the mechanisms that connect the remodeling of the gut microbiota and the development of cardiovascular diseases within the context of the low-pressure and hypoxic environment at high altitudes. At present, the existing evidence shows that the gut microbiota, which acts as a crucial interface linking environmental stress and host physiological homeostasis, plays a dual regulatory role in the process of high-altitude adaptation. The ecological imbalance caused by acute high-altitude exposure, which shows as fluctuations in α-diversity, significant separation in β-diversity, and changes in the abundance of key functional bacterial genera, drives cardiovascular pathological processes through the “gut-heart axis.” Specifically, during acute high-altitude exposure, several core bacterial strains decrease, together with metabolic disturbances like lactate and bile acids, TMAO accumulation, and LPS-derived metabolites entering the circulation, all of which together form the molecular basis of myocardial energy metabolism reprogramming, systemic inflammation activation, and vascular endothelial dysfunction. When the host’s adaptability goes beyond the compensation threshold, such a microbial community imbalance will turn into a pathological state, promoting the occurrence and development of conditions such as atherosclerosis, hypertension, and heart failure.

However, the microbial remodeling observed during the long-term high-altitude adaptation process shows the plasticity as well as the protective potential of the microbiome. Among the guts of high-altitude natives and long-term migrants, probiotics like Prevotella, *Akkermansia muciniphila*, and SCFA-producing bacteria become abundant, accompanied by increased production of SCFAs and reprogramming of purine metabolism, providing metabolic protection to the cardiovascular system by enhancing intestinal barrier integrity, regulating immune balance, and optimizing myocardial energy substrate utilization. This transition from pathological dysregulation to adaptive homeostasis lays a scientific foundation for preventing and treating cardiovascular diseases targeting the gut microbiota in high-altitude areas. Based on this situation, dietary intervention strategies that aim at microbial composition, probiotic/prebiotic supplementation, FMT, and metabolite-targeted regulation, all of which have shown clinical potential in improving hypoxic tolerance and reducing cardiac workload, provide a theoretical foundation for developing new biological interventions in high-altitude medicine.

### 6.2 Research gaps and future directions

Although there has been a fairly large accumulation of experimental and clinical evidence in the current literature, several rather evident methodological limitations and knowledge gaps still exist. First, the existing research has a lack of standardization regarding altitude definition, cohort type, and exposure duration. Furthermore, inadequate control of composite environmental variables, along with dietary habits and host genetic background, makes it quite difficult to accurately differentiate the independent impact of hypoxia and the interaction of confounding factors. More importantly, current microbiome research mainly depends on the relative abundance analysis that is based on the 16S rRNA gene sequencing. However, this method has an inherent limitation: since the data has a “combinatorial” nature, any change in the relative proportion of any single taxonomic unit has to depend on the proportions of all the other taxonomic units in the community. When there are differences in total microbial load among samples, relative abundance analysis is particularly prone to misunderstanding. For example, in a high-altitude hypoxic environment, one can observe that the total microbial load in the intestine is significantly reduced. In such circumstances, the increase in the relative abundance of a particular genus may not actually signify that its absolute abundance has truly increased, but instead might be a passive relative increase resulting from the decrease in the absolute abundance of other microbial populations (Barlow et al., 2020). Additionally, the taxonomic discrimination ability of the 16S technique only reaches the genus level, which makes it difficult to identify the functional heterogeneity of specific strains and their direct causal relationships with cardiovascular phenotypes. For example, as with the previously mentioned Prevotella enrichment phenomenon, whether it is Prevotella-dominated SCFA metabolism that promotes cardiovascular adaptation or whether it is lipopolysaccharide released by specific strains in the Prevotella genus (such as *Prevotella copri*) that exacerbates inflammation risk, this still needs confirmation through gnotobiotic animal colonization experiments and prospective intervention studies. Meanwhile, the practical problems in clinical practice, such as the criteria for the selection of probiotic strains, the matching principles of donors for FMT, and interactions with commonly used high-altitude drugs, still lack large-scale, rigorously designed randomized controlled trials (RCTs) to verify their long-term safety and efficacy.

Future research should focus on overcoming the above limitations. Methodologically, it is recommended to introduce techniques such as Quantitative Microbiome Profiling (QMP) to obtain absolute abundance data of microbial taxa for correcting relative abundance biases, and to establish a unified high-altitude exposure assessment system (Vandeputte et al., 2017). Meanwhile, constructing a deeply phenotyped multi-omics longitudinal cohort is also crucial by adopting a comprehensive strategy integrating metagenomics, metabolomics, and host transcriptomics, to overcome the technical limitations of 16S rRNA sequencing. Particularly, the technique of metagenomic genome assembly is to be employed in order to identify the core genome specific to the high-altitude population, which contributes to the identification of the key functional gene cluster that determines the hypoxia tolerance ability (Broeksema et al., 2017). The precise intervention based on the mechanism and the whole life cycle management will be the core direction in the future. Future prevention and treatment strategies should shift from macro-level dietary recommendations to micro-level microbiota reshaping, targeting key pathological links such as insufficient SCFA production, accumulation of TMAO, and bile acid metabolic disorders mentioned earlier. Compared to FMT with complex components, which involves complex components, the construction of clear synthetic microbial communities is becoming a research hotspot of the next-generation microbial therapy due to controllable safety and clear mechanisms (Sahle et al., 2024). For example, creating synthetic communities imitating the metabolic characteristics of plateau residents, or developing engineered strains specifically regulating bile acid metabolism, such as new approaches that can overcome the current treatment bottlenecks, are quite promising. Furthermore, since high-altitude adaptation is a dynamic process, the research scope should be expanded to include the entire life cycle and intergenerational transmission. According to the “Developmental Origins of Health and Disease” (DOHaD) theory, what needs to be studied is the way that the maternal microbiota programs cardiovascular adaptability in offspring through such approaches as human milk oligosaccharides (HMOs) (Stiemsma and Michels, 2018). Ultimately, by establishing a multi-dimensional prediction model which integrates the host’s genotypes, microbiota characteristics, metabolic phenotypes, and then by using machine learning algorithms to predict the individual’s response rate to interventions, we can achieve a shift in the transformation from passive adaptation to active regulation, thereby being able to provide a precision microbiota-based solution for cardiovascular health in high-altitude regions.

In summary, research on the gut microbiota is undergoing a profound transformation, shifting from descriptive associations to mechanistic explanations and precise interventions. Through the integration of interdisciplinary disciplines together with clinical verification, a new paradigm based on microbiome regulation to prevent and treat cardiovascular diseases at high altitude has been developed, which will ultimately enhance the health adaptability of residents and travelers in global high-altitude regions.
